# Flurbiprofen ameliorated obesity by attenuating leptin resistance induced by endoplasmic reticulum stress

**DOI:** 10.1002/emmm.201303227

**Published:** 2014-01-14

**Authors:** Toru Hosoi, Rie Yamaguchi, Kikuko Noji, Suguru Matsuo, Sachiko Baba, Keisuke Toyoda, Takahiro Suezawa, Takaaki Kayano, Shinpei Tanaka, Koichiro Ozawa

**Affiliations:** 1 Department of Pharmacotherapy, Graduate School of Biomedical and Health Sciences, Hiroshima UniversityHiroshima, Japan; 2 School of Integrated Arts ' Sciences, Hiroshima UniversityHigashi-Hiroshima, Japan

**Keywords:** STAT3, aldehyde dehydrogenase, nonsteroidal anti-inflammatory drug

## Abstract

Endoplasmic reticulum (ER) stress, caused by the accumulation of unfolded proteins, is involved in the development of obesity. We demonstrated that flurbiprofen, a nonsteroidal anti-inflammatory drug (NSAID), exhibited chaperone activity, which reduced protein aggregation and alleviated ER stress-induced leptin resistance, characterized by insensitivity to the actions of the anti-obesity hormone leptin. This result was further supported by flurbiprofen attenuating high-fat diet-induced obesity in mice. The other NSAIDs tested did not exhibit such effects, which suggested that this anti-obesity action is mediated independent of NSAIDs. Using ferriteglycidyl methacrylate beads, we identified aldehyde dehydrogenase as the target of flurbiprofen, but not of the other NSAIDs. These results suggest that flurbiprofen may have unique pharmacological properties that reduce the accumulation of unfolded proteins and may represent a new class of drug for the fundamental treatment of obesity.

**Subject Categories** Metabolism; Pharmacology & Drug Discovery

## Introduction

Obesity has become a serious global health concern. However, most pharmacological treatments for obesity are based on symptoms and effective medications are lacking. The mechanisms of obesity began to be eluciated after the anti-obesity hormone leptin was identified (Zhang *et al*, 1994).

The leptin receptor (Ob-Rb) has an amino acid sequence that is involved in the activation of janus kinase 2-signal transducer and activator of transcription 3 (JAK2-STAT3) tyrosine kinases. Leptin was previously shown to activate JAK2, which subsequently phosphorylated Tyr1138 residues within Ob-Rb (Li ' Friedman, 1999). Phosphorylated Tyr1138 has also been shown to activate STAT3 (Banks *et al*, 2000). A previous study demonstrated that leptin was mainly secreted from adipose tissue and circulated in the blood stream (Morton ' Schwartz, 2011). Circulating leptin inhibits food intake by activating Ob-Rb expressed on the hypothalamus (Campfield *et al*, 1995; Vaisse *et al*, 1996; Hosoi *et al*, 2002). The essential role of the Ob-Rb-STAT3 signal was demonstrated by replacing Tyr 1138 in Ob-Rb with a serine (S1138), which disrupted the Ob-Rb-STAT3 signal, resulting in obesity (Bates *et al*, 2003).

Since leptin exerts an anti-obesity effect, it was initially expected to be an anti-obesity drug. However, its effect was modest in obese patients (Mantzoros ' Flier, 2000), most of whom were considered to be in a state of leptin resistance and unable to adequately respond to the anti-obesity signals of leptin (Münzberg ' Myers, 2005). Therefore, leptin resistance is a major factor in the development of obesity (Friedman, 2003). Several important findings have been reported regarding the mechanisms of leptin resistance. Suppressor of cytokine signaling 3 (SOCS3) (Bjørbæk *et al*, 1998) and protein tyrosine phosphatase 1B (PTP1B) (Cheng *et al*, 2002; Zabolotny *et al*, 2002) have been implicated in the development of leptin resistance. Meanwhile, a recent study suggested that endoplasmic reticulum (ER) stress may be involved in the development of obesity (Ozcan *et al*, 2004). The ER is an organelle involved in the folding of new proteins. However, stressors that disrupt ER function can cause the accumulation of unfolded proteins (Tabas ' Ron, 2011; Walter ' Ron, 2011). Therefore, reducing the amount of unfolded proteins using a pharmacological approach may represent a promising fundamental treatment for this disease. Based on these findings, we attempted to identify a new drug that can ameliorate obesity. In the present study, we demonstrated that flurbiprofen could be used a candidate drug to ameliorate obesity.

Flurbiprofen is a NSAID that effectively treats pain, inflammation, and fever. NSAIDs are drugs that can inhibit cyclooxygenase (COX), which is responsible for converting arachidonic acid to prostaglandins. Flurbiprofen has been classified as the 2-arylpropionic acid of NSAIDs. It is currently widely prescribed for arthritis, rheumatism, and osteoarthritis, and is used to treat the inflammation and pain associated with these diseases. However, to the best of our knowledge, the pharmacological effect of flurbiprofen on obesity has not yet been determined.

## Results

### Flurbiprofen attenuated protein aggregation

**Figure 1:**
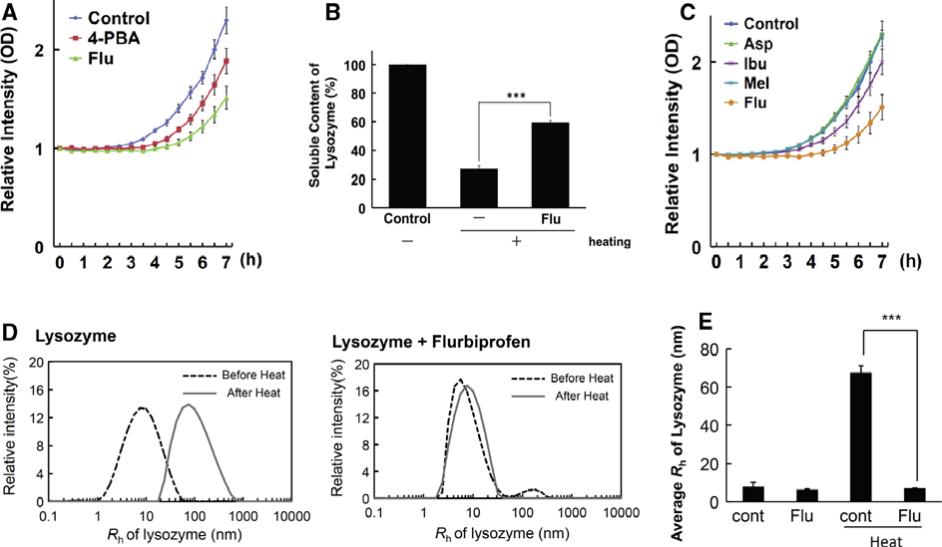
Flurbiprofen inhibited protein aggregation.
Chaperone activity of flurbiprofen (Flu). The rate of aggregation of reduced-α-lactalbumin (r-LA) was measured in the presence or absence of drugs. The aggregation of r-LA was induced by BSA aggregates. Aggregation was monitored by measuring its turbidity at 488 nm. Aggregation gradually increased in a time-dependent manner following the addition of denatured BSA (0–7 h at 37°C). Flurbiprofen (100 μM) exhibited stronger chaperone activity than that of sodium 4-phenylbutyrate (4-PBA, 100 μM), which was used as a positive control *n* = 6.The heat-induced aggregation of lysozyme was measured. Flurbiprofen inhibited heat-induced aggregation *n* = 6.Chaperone activities of the other NSAIDs (Asp: aspirin, Ibu: ibuprofen, Mel: meloxicam) at 100 μM. The rate of aggregation of r-LA was measured in the presence or absence of drugs, as was also performed in (A). Ibuprofen exhibited weak activity, while the other NSAIDs showed none *n* = 5–7.Intensity-weighted size distributions of lysozyme aggregates measured by DLS. Heating at 42°C for 5 min shifted *R*_h_ to a high value for the lysozyme sample, whereas it did not for the flurbiprofen + lysozyme sample. ^***^P < 0.001, *n* = 6.Average *R*_h_ of lysozyme measured by DLS. Heat-induced increases in lysozyme *R*_h_ were significantly inhibited by flurbiprofen.

Flurbiprofen is known to inhibit the progression of Alzheimer's disease (Eriksen *et al*, 2003), which is caused by ER stress (Nakagawa *et al*, 2000); therefore, we speculated that flurbiprofen may be able to attenuate ER stress. To test this hypothesis, we investigated whether flurbiprofen could reduce protein aggregation and function as a chemical chaperone. Chemical chaperones are a group of low-molecular weight compounds that can correct unfolded/aggregated proteins (Perlmutter, 2002). We measured the aggregation of denatured lactalbumin in an *in vitro* system. We observed the chaperone activity of flurbiprofen and found that it markedly attenuated protein aggregation. This effect was stronger than that of 4-phenylbutyrate (4-PBA), which was used as a positive control (Kubota *et al*, 2006; Ozcan *et al*, 2009) (Fig 1A). To further confirm these results, we measured the heat-induced aggregation of lysozyme in another system to assess chaperone activity. Heating lysozyme led to aggregation (Fig 1B). However, when flurbiprofen was added, we observed a significant 2-fold increase in the soluble protein concentration (Fig 1B). We then performed dynamic light scattering (DLS) analysis using lysozyme to determine whether flurubiprofen inhibited protein aggregation. The heat-induced aggregation of lysozyme increased the average hydrodynamic radius (*R*_h_) from *R*_h_ = 7.9 to *R*_h_ = 67.6, and this was significantly inhibited by the addition of flurbiprofen (Fig 1E). Typical DLS data of the intensity-weighted size distributions of lysozyme aggregates obtained by DLS are shown in Fig 1D. These results suggest that flurbiprofen can attenuate the formation of lysozyme aggregats.

Since flurbiprofen is a NSAID, we examined whether other NSAIDs also exhibited chaperone activity. We measured the effects of various NSAIDs on the aggregation of denatured lactalbumin *in vitro*. Aspirin and meloxicam did not exhibit chaperone activity, while that of ibuprofen was weak (Fig 1C). These results demonstrate that flurbiprofen is different from the other NSAIDs because it exhibits strong chaperone activity.

**Figure 2:**
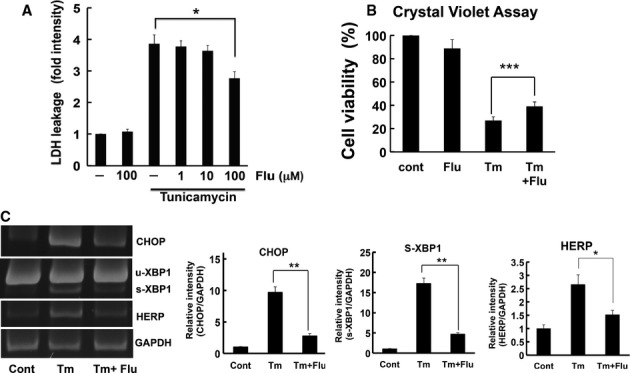
Flurbiprofen alleviated ER stress.
Flurbiprofen inhibited ER stress-induced cell death. SH-SY5Y cells were treated with tunicamycin (Tm: 1 μg/ml) in the presence or absence of flurbiprofen (Flu) for 48 h. LDH activity was measured as an indicator of cytotoxicity. *P <* *0.05, *n* = 3–4.SH-SY5Y cells were treated with tunicamycin (Tm: 1 μg/ml, 48 h) and cell viability was analyzed by the crystal violet assay. Flurbiprofen (Flu) increased cell viability. ^***^P <* *0.001 *n* = 5.Flurbiprofen (Flu: 100 μM) inhibited UPR in SH-SY5Y cells. The induction of CHOP, XBP1 splicing, and HERP was analyzed by RT-PCR, *n* = 3–4.

Protein folding is known to attenuate ER stress (Kim *et al*, 2008). Thus, we investigated whether flurbiprofen inhibited ER stress-induced cell death to determine its pharmacological properties in a cellular system. The SH-SY5Y neuroblastoma cell line was treated with flurbiprofen and ER stress-induced cell death was analyzed by measuring the activity of LDH. ER stress (tunicamycin or brefeldin A)-induced cell death was significantly inhibited by flurbiprofen (Fig 2A and supplementary Fig S1). Flurbiprofen also significantly improved cell viability, as confirmed by the crystal violet assay (Fig 2B). Moreover, flurbiprofen inhibited the ER stress-induced activation of the unfolded protein response (UPR). ER stress (tunicamycin) activated three branches of the UPR; the PERK, IRE1α, and ATF6 branches, as indicated by the induction of CHOP transcription (Harding *et al*, 2000), XBP1 splicing (Yoshida *et al*, 2001; Calfon *et al*, 2002), and HERP expression (Kokame *et al*, 2001; Yamamoto *et al*, 2007), respectively, in the SH-SY5Y neuroblastoma cell line (Fig 2C). The treatment with flurbiprofen significantly inhibited all three branches of the UPR (Fig 2C). Taken together, these results demonstrate that flurbiprofen has the ability to reduce ER stress.

### Flurbiprofen attenuated leptin resistance

**Figure 3:**
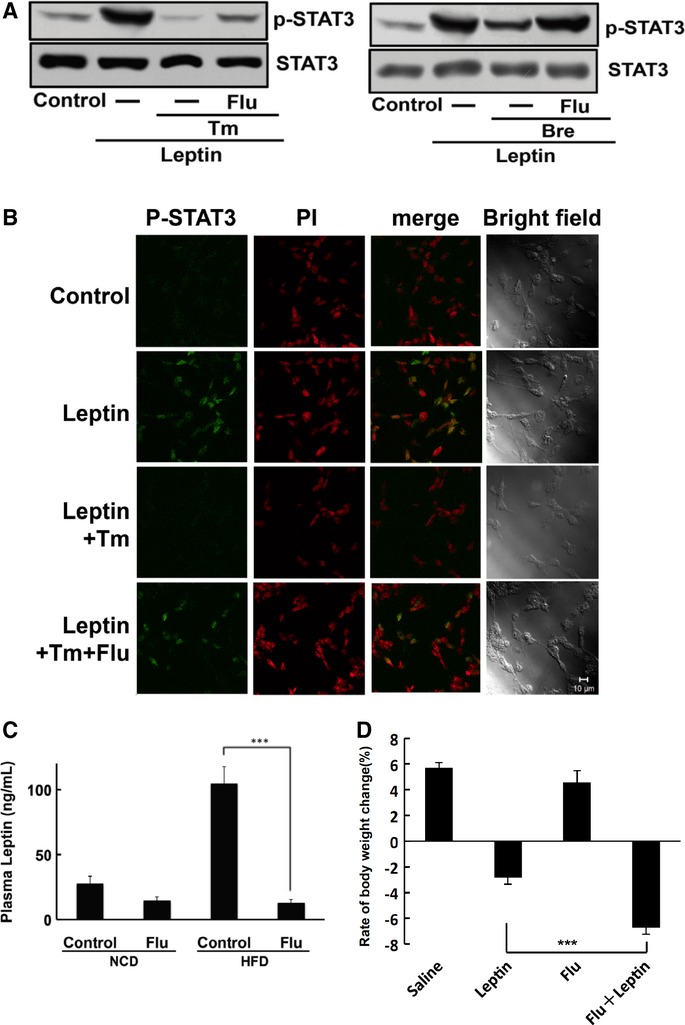
Flurbiprofen attenuated leptin resistance.
Flurbiprofen reversed ER stress-induced leptin resistance. Leptin-induced STAT3 activation was inhibited by ER stress and this inhibitory effect was ameliorated by flurbiprofen. Tm: Tunicamycin; Bre: Brefeldin A.Flurbiprofen reversed the ER stress-induced attenuation of nuclear phospho-STAT3 staining, caused by leptin. PI: Propidium iodide. Scale bar, 10 μM.Flurbiprofen inhibited the HFD-induced elevation in circulating leptin levels. Mice were concomitantly fed a normal chow diet (NCD) or HFD with or without flurbiprofen (Flu) for 8 weeks. *n* = 7–8 per group. ^***^P <* *0.001 versus high-fat diet.ob/ob mice were treated with flurbiprofen (Flu) in combination with leptin and body weight was analyzed. Data were expressed as the rate of body weight change (%), which was compared between day 1 and day 7. Flurbiprofen significantly enhanced the effects of leptin on body weight reduction. ^***^P <* *0.001 versus the leptin treatment alone, *n* = 9–12. Source data are available for this figure.

ER stress is known to be involved in the development of leptin resistance (Hosoi *et al*, 2008; Zhang *et al*, 2008; Ozcan *et al*, 2009; Won *et al*, 2009). Because flurbiprofen was previously shown to attenuate ER stress, we investigated whether it attenuated leptin resistance. We examined whether flurbiprofen attenuated the ER stress-induced impairment in leptin signaling; i.e. STAT3 activation (Vaisse *et al*, 1996; Hosoi *et al*, 2008; Ozcan *et al*, 2009). ER stress impaired leptin-induced STAT3 activation and this effect was reversed by flurbiprofen in the SH-SY5Y-Ob-Rb neuroblastoma cell line (Fig 3A). The ER stress-induced impairment in nuclear P-STAT3 staining was also reversed by flurbiprofen (Fig 3B). Therefore, these results suggest that flurbiprofen can attenuate ER stress-induced leptin resistance. Plasma leptin levels have been strongly correlated with body mass index (BMI) (Maffei *et al*, 1995). High circulating leptin levels are used as a physiological indicator of leptin resistance. Accordingly, we measured circulating leptin levels to evaluate the efficiency with which flurbiprofen reduced leptin resistance *in vivo*. Circulating leptin levels were increased in mice fed a high-fat diet for 8 weeks (Fig 3C). However, these levels normalized and were similar to those in the normal-chow diet-fed group following treatment with flurbiprofen in the fed (Fig 3C) or fasted (supplementary Fig S2) state. We further investigated the effects of flurbiprofen on body weight loss using ob/ob mice, another model of obesity, which have no functional leptin (Zhang *et al*, 1994) and were previously shown to be in a state of ER stress (Ozcan *et al*, 2004). We injected recombinant leptin with flurbiprofen and measured body weight. The treatment with leptin alone decreased body weight, and responsiveness to the actions of leptin was further increased by flurbiprofen (Fig 3D). In contrast, flurbiprofen alone only had a slight effect on body weight. These results suggest that flurbiprofen has the ability to increase the sensitivity of the actions of leptin at the level of physiological output i.e. the inhibitory effect of leptin on body weight. Food intake was also significantly inhibited in leptin + flurbiprofen-treated mice (supplementary Fig S3). Accordingly, our *in vitro* and *in vivo* results indicate that flurbiprofen may be able to attenuate leptin resistance and increase sensitivity to the actions of leptin.

### Anti-obesity effect of flurbiprofen

**Figure 4:**
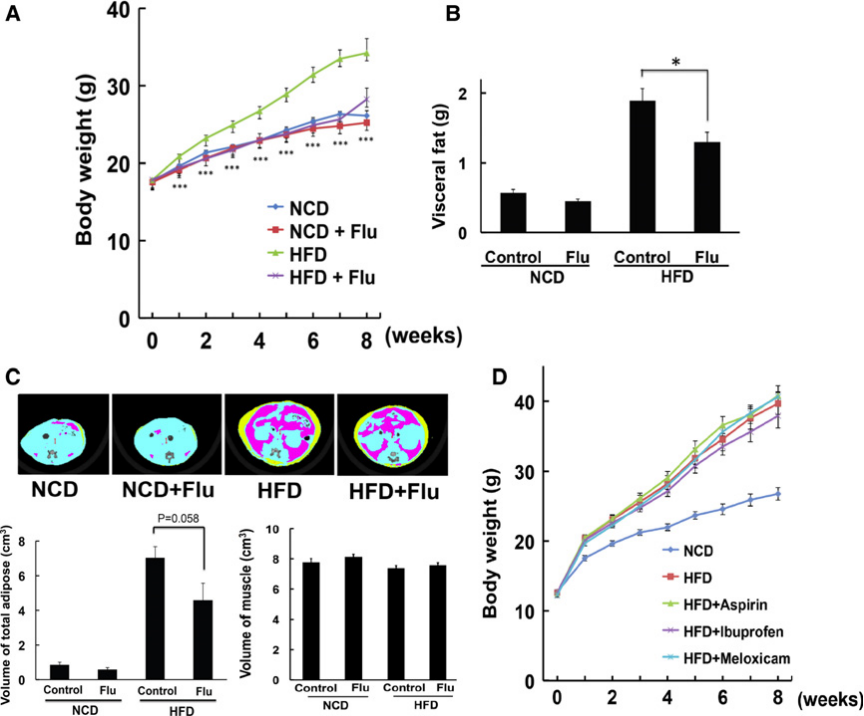
Anti-obesity effect of flurbiprofen.
Flurbiprofen reduced HFD-induced body weight gain. Body weight was measured in HFD-fed or control-diet-fed mice treated with flurbiprofen (10 mg/kg/day). *n* = 15, ^***^P <* *0.001 versus HFD.Flurbiprofen inhibited the HFD-induced increase in visceral fat. The total weight of visceral fat was measured in each mouse 8 weeks after the treatments. *n* = 7–8 per group. *P <* *0.05 versus HFD.Effect of flurbiprofen on fat accumulation assessed using CT scans. Yellow indicates subcutaneous adipose tissue and red indicates visceral adipose tissue. Flurbiprofen inhibited the HFD-induced increase in adipose volume, but not muscle volume. *n* = 7.Effect of the other NSAIDs, aspirin (Asp: 10 mg/kg/day), meloxicam (Mel: 1 mg/kg/day), and ibuprofen (Ibu: 60 mg/kg/day), on HFD-induced body weight gain. These NSAIDs did not have an anti-obesity effect. *n* = 8.

We next determined whether flurbiprofen had an anti-obesity effect. Four-week-old mice were fed a high-fat diet (HFD) for 8 weeks (Fig 4A). Flurbiprofen was simultaneously administered with the HFD and body weight was measured. The HFD increased body weight and this increase was markedly attenuated by the treatment with flurbiprofen (Fig 4A). This weight-reducing effect was not observed in control mice on a normal diet, which indicated that this effect was specific to HFD-induced weight gain (Fig 4A). Measurements revealed that flurbiprofen decreased visceral fat weight (Fig 4B). Computed tomography (CT) showed that the accumulation of fat (viscera and subcutaneous fat) was inhibited in the flurbiprofen-treated group (Fig 4C, supplementary Fig S4). On the other hand, viscera muscle volume was not affected by the treatment, suggesting that this effect was specific (Fig 4C). No significant differences were observed in locomotor activity among the control- versus flurbiprofen-treated groups, which demonstrated that the results obtained were not due to side-effects (Supplementary Fig S5). In addition, no significant difference was observed in body length between HFD versus HFD + flurbiprofen fed mice; therefore, flurbiprofen did not affect body size (supplementary Fig S6). We next investigated whether other NSAIDs exhibited similar properties. Aspirin, ibuprofen, and meloxicam were administered simultaneously with the HFD and body weight was measured. We did not observe a significant weight-reducing effect by these drugs (Fig 4D). Taken together, these results suggest that flurbiprofen has an anti-obesity effect that is mediated through a novel mechanism (chemical chaperone activity) independent of NSAID activity.

### Identification of flurbiprofen-interacting proteins

**Figure 5:**
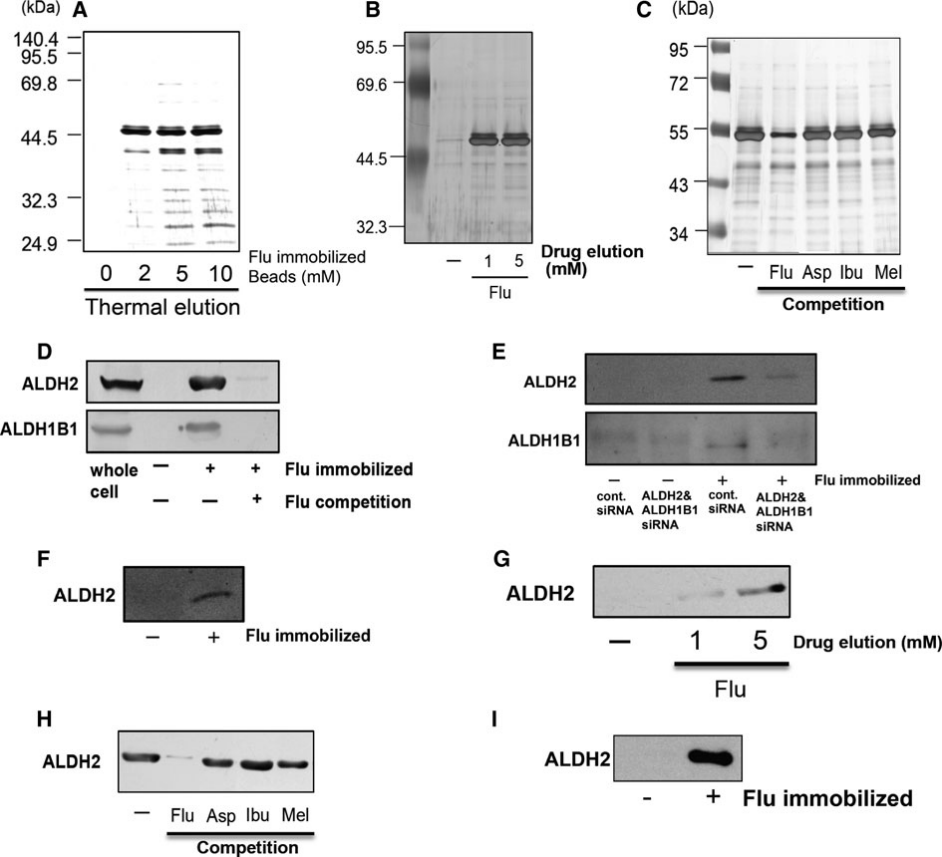
Identification of flurbiprofen-interacting proteins..
Flurbiprofen-binding proteins were purified from mouse liver samples. Eluted proteins were analyzed by silver staining.Flurbiprofen-immobilized beads were incubated with mouse liver lysates. Flurbiprofen-bound proteins were then eluted with an excess amount of flurbiprofen.Excess amounts of flurbiprofen, aspirin, ibuprofen, and meloxicam (0.2 mM) were added to the extracts before incubation with the beads. Pre-incubating the lysates with flurbiprofen reduced the amount of the 50-kDa bands, while pre-incubating lysates with other NSAIDs did not.Aldehyde dehydrogenases (ALDH2 and ALDH1B1) were detected in the eluted sample obtained from flurbiprofen-immobilized beads by Western blotting. Pre treatment with an excess amount of flurbiprofen (0.2 mM) before incubation with the FG beads resulted in a decrease in the yields of these proteins. The whole cell lysate of a mouse liver sample was used as a positive control.Flurbiprofen-immobilized beads were incubated with HEK293T cells, which were silenced with ALDH2/ALDH1B1 siRNAs. Thermal-eluted proteins were analyzed by Western blotting.Flurbiprofen-immobilized beads were incubated with the lysate of the SH-SY5Y cell line. Thermal-eluted proteins from the beads were analyzed by Western blotting.Flurbiprofen-eluted samples, as shown in Fig 4B, were subjected to Western blotting.Excess amounts of flurbiprofen, aspirin, ibuprofen, and meloxicam (0.2 mM) were added to the extracts before incubation with the beads. The eluted samples were subjected to Western blotting.Direct interaction of ALDH2 with flurbiprofen. Human recombinant ALDH2 was incubated with flurbiprofen-immobilized beads, and ALDH2 was detected by Western blotting. Source data are available for this figure.

Flurbiprofen is known to have unique pharmacological properties that attenuate obesity differently from the other NSAIDs; therefore, we attempted to identify flurbiprofen-interacting proteins. We performed affinity purification using ferriteglycidyl methacrylate (FG) beads (Shimizu *et al*, 2000). The derivative of flurbiprofen, 4′-hydroxy flurbiprofen, was covalently coupled with epoxy FG beads (supplementary Fig S7) and incubated with the lysate of a mouse liver. We obtained a major band approximately 50 kDa from the sample obtained by thermal elution (Fig 5A). At the same time, the yields of the band decreased when flurbiprofen was added to the extracts before incubation with the beads, which confirmed its specificity (Fig 5C). We performed drug elution to re-confirm this specificity, and the 50-kDa band was detected in the eluted sample (Fig 5B). To determine whether this protein interacted with the other NSAIDs, liver extracts were pre-incubated with aspirin, ibuprofen, and meloxicam before the sample was incubated with flurbiprofen-coupled beads. Although the pre-incubation with flurbiprofen reduced the amount of the 50-kDa band, that with aspirin, ibuprofen, or meloxicam did not (Fig 5C). Therefore, the 50-kDa protein appears to interact with flurbiprofen, but not with the other NSAIDs. Flurbiprofen-interacting proteins were also observed in other tissues (supplementary Fig S8). The 50-kDa bands were then subjected to nano LC-MS/MS and identified as aldehyde dehydrogenase, mitochondrial (ALDH2), and aldehyde dehydrogenase family 1 member B1 (ALDH1B1). The identities of these proteins were confirmed by Western blotting (Fig 5D). Furthermore, silencing ALDH2/ALDH1B1 by its siRNAs in HEK293T cells resulted in a reduction in flurbiprofen-interacting proteins, which confirmed its specificity (Fig 5E). The interaction of these proteins with flurbiprofen was also detected in the neuronal SH-SY5Y cell line (Fig 5F). Flurbiprofen-eluted samples were also confirmed to be ALDH2 by Western blotting (Fig 5G). The results of competition analysis indicated that ALDH2 interacted with flurbiprofen, but not with the other NSAIDs (Fig 5H). Finally, a direct interaction was confirmed by incubating a recombinant ALDH2 protein with flurbiprofen-coupled beads (Fig 5I). Taken together, these findings indicate that flurbiprofen can interact with aldehyde dehydrogenase.

**Figure 6:**
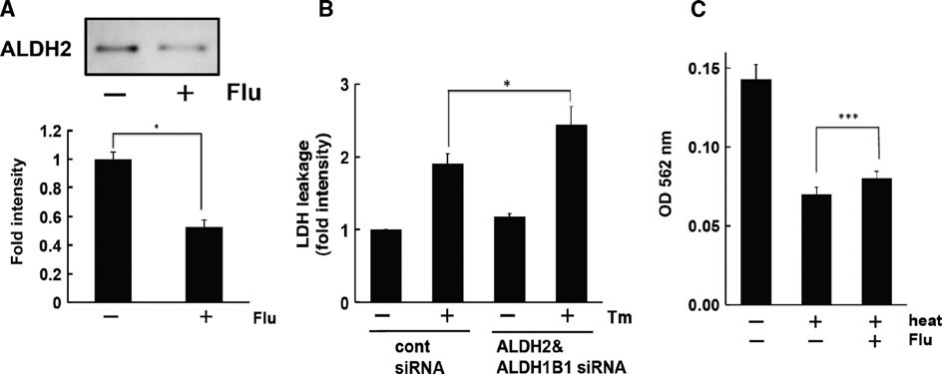
Aldehyde dehydrogenase interacted with flurbiprofen and attenuated ER stress-induced cell death.
Interaction of ALDH2 with flurbiprofen under physiological conditions. HEK 293 cells were treated with flurbiprofen (100 μM, 4 h) and lysates were harvested. The lysates were incubated with flurbiprofen-immobilized beads and bound protein was analyzed by Western blotting. ^*^P <* *0.05, *n *=* *3.ER stress-induced cell death was enhanced by knocking down ALDH2 and ALDH1B1. SH-SY5Y cells were treated with tunicamycin (Tm: 1 μg/ml) for 24 h and LDH activity was measured. ^*^P <* *0.05, *n* = 7.Flurbiprofen inhibited the aggregation of ALDH2. The heat-induced aggregation of ALDH2 was analyzed in the presence of flurbiprofen (30 mM). ^***^P <* *0.001, *n* = 6. Source data are available for this figure.

**Figure 7:**
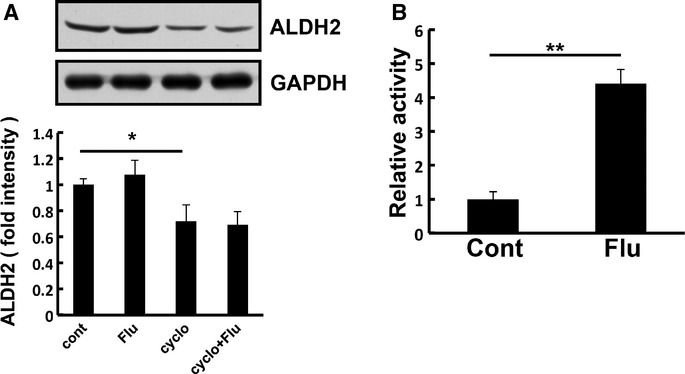
Effects of flurbiprofen on ALDH2 expression, stability, and activity.
HEK293T cells were treated with flurbiprofen in the presence or absence of cycloheximide (CHX: 25 μg/ml) for 24 h and the expression level of ALDH2 was analyzed. Flurbiprofen did not affect the expression or stability of ALDH2.Aldehyde dehydrogenase activity was measured in the presence or absence of flurbiprofen (100 μM) in HEK293-Ob-Rb cells. Flurbiprofen increased aldehyde dehydrogenase activity. Source data are available for this figure.

To confirm whether aldehyde dehydrogenase interacted with flurbiprofen in living cells, cultured cells (HEK293 cells) were treated with flurbiprofen for 4 h and then lysed and incubated with flurbiprofen-coupled beads. The bound proteins were eluted from the beads and subjected to Western blotting. ALDH2 was detected in the eluted sample from cells, and the band was significantly decreased by the flurbiprofen treatment (Fig 6A). This decrease may have been due to a reduction in the free form of ALDH2, which can interact with flurbiprofen. Therefore, flurbiprofen may interact with ALDH2 in living cells. The knock down of aldehyde dehydrogenase in SH-SY5Y Ob-Rb cells enhanced ER stress-induced cell death (Fig 6B and supplementary Fig S9). Thus, aldehyde dehydrogenase may function to resist ER stress. Furthermore, the heat-induced aggregation of ALDH2 was significantly inhibited by flurbiprofen (Fig 6C), which supported the hypothesis that flurbiprofen can attenuate the accumulation of unfolded proteins, thereby reducing ER stress. We analyzed ALDH2 expression levels after cells were treated with flurbiprofen and found that they were not affected (Fig 7A). We also analyzed the stability of ALDH2 after the treatment with flurbiprofen. We blocked protein translation by treating cells with cycloheximide (CHX) and analyzed the effect of flurbiprofen on its expression. The treatment with CHX reduced ALDH2 levels (Fig 7A). However, flurbiprofen did not affect the expression level of ALDH2 following the CHX treatment (Fig 7A), which suggests that flurbiprofen may not affect ALDH2 stability. On the other hand, we measured the effect of flurbiprofen on the activity of aldehyde dehydrogenase and demonstrated that it was increased by flurbiprofen (Fig 7B).

**Figure 8:**
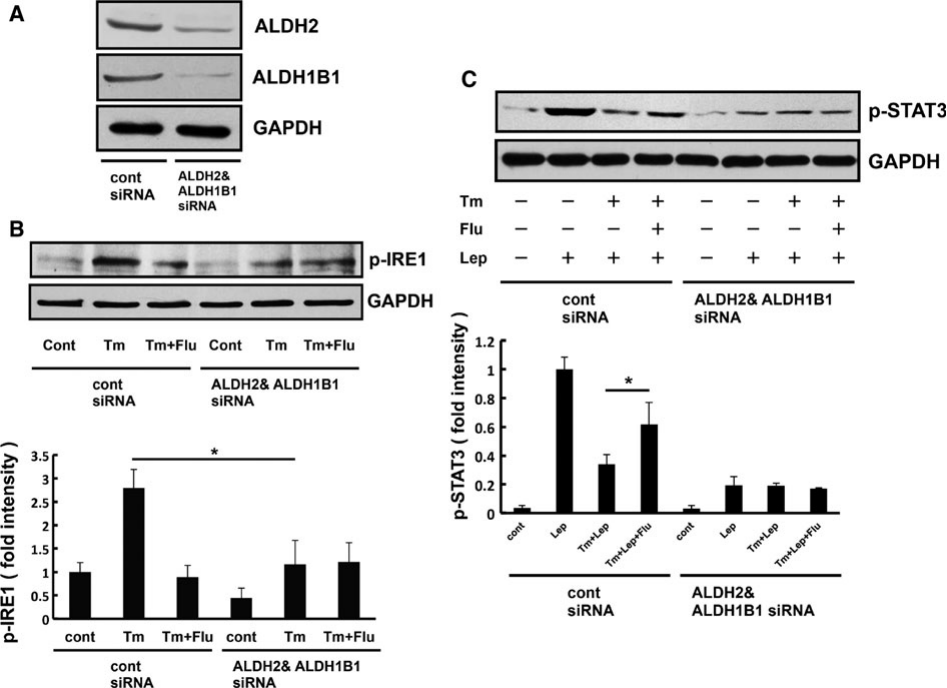
Pharmacological effect of flurbiprofen in aldehyde dehydrogenase knocked-down cells.
Western blot analysis of ALDH2/ALDH1B1expression in the lysates of cells transfected with siRNAs directed at ALDH2/ALDH1B1 or the control sequence. The expression of ALDH2/ALDH1B1 with ALDH2/ALDH1B1 siRNA than with control siRNA.ER stress-induced IRE1 phosphorylation was analyzed in HEK293-Ob-Rb cells. These cells were treated with tunicamycin (Tm: 0.3 μg/ml) and flurbiprofen (Flu: 100 μM) for 4 h and Western blotting was then performed. The knock down of ALDH2/ALDH1B1 attenuated UPR. Flurbiprofen could not ameliorate the ER stress-induced activation of IRE1 in ALDH2/ALDH1B1 knocked-down cells.HEK293-Ob-Rb cells were treated with tunicamycin (Tm: 0.3 μg/ml) and flurbiprofen (Flu: 100 μM) for 4 h and leptin-induced STAT3 phosphorylation was analyzed. Flurbiprofen did not ameliorate ER stress-induced leptin resistance in ALDH2/ALDH1B1 knocked-down cells. Source data are available for this figure.

We then examined whether aldehyde dehydrogenase silencing had any effect on UPR and leptin-induced STAT3 signaling. We found that ER stress-induced IRE1 phosphorylation was significantly reduced in aldehyde dehydrogenase knocked-down cells (Fig 8A and B). Flurbiprofen did not further attenuate ER stress-induced IRE1 phosphorylation in the knocked-down cells (Fig 8B). Therefore, the aggregated form of aldehyde dehydrogenase is involved in activating UPR and flurbiprofen may attenuate ER stress by reducing its aggregation. Leptin-induced STAT3 phosphorylation was attenuated by its knock down (Fig 8C). The pharmacological effect of flurbiprofen on the attenuation of leptin-induced STAT3 phosphorylation in ER-stressed cells was lost by knocking down aldehyde dehydrogenase (Fig 8C).

## Discussion

Flurbiprofen is a NSAID that can inhibit cyclooxygenase activity. In the present study, we demonstrated that flurbiprofen had an anti-obesity effect that was not achieved simply through its role as a NSAID. This result is consistent with the previous finding in which aspirin, an NSAID, had no anti-obesity effect (Yuan *et al*, 2001). Thus, the inhibition of cyclooxygenase activity may not have an anti-obesity effect. What is the underlying mechanism for the action of flurbiprofen? Because chronic metabolic diseases disrupt the folding capacity of multiple proteins, we hypothesized that flurbiprofen had the unique effect of promoting folding. We showed that flurbiprofen exhibited strong chaperone activity, which assisted protein folding. Therefore, the therapeutic properties of flurbiprofen may partly be achieved through a protein-stabilizing action, which subsequently attenuates ER stress.

We then examined the mechanisms of this pharmacological action at the molecular level. We searched for proteins that interacted with flurbiprofen. Using FG-beads and nano LC-MS/MS analysis, we identified these proteins as aldehyde dehydrogenases. Alcohol ingestion has been shown to elevate acetaldehyde levels, the oxidized metabolite of ethanol. Aldehyde dehydrogenases are enzymes involved in catalyzing the oxidation of aldehydes to produce carboxylic acids. Aldehyde dehydrogenase activity was reported to be increased in patients with non-alcoholic steatohepatitis (NASH), which was suggested to be associated with obesity (Baker *et al*, 2010). Thus, flurbiprofen may be useful in the treatment of NASH due to its effects on ALDH2 function because the detoxication of aldehydes may be beneficial in this treatment. ER stress was suggested to be associated with NASH (Rahman *et al*, 2007; Puri *et al*, 2008). Aldehyde dehydrogenase has been shown to participate in the removal of 4-hydroxynonenal (4-HNE), which causes ER stress (West ' Marnett, 2005). A deficiency in aldehyde dehydrogenase activity was reported to increase vulnerability to 4-HNE (Ohsawa *et al*, 2003). An increased amount of 4-HNE has also been suggested to be associated with obesity and diabetes (Suarez-Pinzon *et al*, 1996; Miwa *et al*, 2000; Toyokuni *et al*, 2000; Mattson, 2009). We observed that the knock down of aldehyde dehydrogenase genes elevated ER stress-induced cell death. Based on the present findings, we hypothesized the pharmacological mechanism for the action of flurbiprofen as follows: Aldehyde dehydrogenase may be aggregated and attenuate leptin signaling under stressed conditions. Flurbiprofen may inhibit this aggregation, thereby attenuating leptin resistance. Therefore, flurbiprofen may reduce the aggregation of aldehyde dehydrogenase, which may, in turn, reduce ER stress and the subsequent development of obesity. On the other hand, because the effects of flurbiprofen on ER stress and leptin signaling were mild, except for that on body weight, we cannot deny the possibility that flurbiprofen may also have other unknown pharmacological properties that reduce body weight. Further investigations on these possibilities are warranted in future studies.

Several anti-obesity drugs can have serious side effects (Cooke ' Bloom, 2006). However, flurbiprofen appears to be safe because it has been used for decades to treat rheumatism, analgesia, and inflammatory disorders. Therefore, the present results may offer a safe treatment for metabolic syndrome. Flurbiprofen was shown to have a protein-stabilizing effect; therefore, it may result in a basic treatment. Thus, the unique pharmacological effects of the classic drug flurbiprofen may offer therapeutic opportunities for the treatment of obesity. Furthermore, our results highlight the possibility of a new approach to the treatment of protein-folding diseases such as diabetes, neurodegenerative diseases, and cancer.

## Materials and Methods

### Reagents

Tunicamycin, 4-phenylbutylate, cycloheximide, ibuprofen, and lysozyme were obtained from Wako Pure Chemical Industries, Ltd. (Osaka, Japan). The ALDH2 protein (18–517 aa) was obtained from ATGen (Seoul, Korea). (±)-Flurbiprofen were from Sigma (St Louis, MO, USA) or Cayman Chemical (Ann Arbor, MI, USA). α-Lactalbumin, aspirin, and meloxicam were from Sigma. Human recombinant leptin for use *in vitro* was from Sigma and mouse recombinant leptin for use *in vivo* was from R'D Systems (Minneapolis, MN, USA). Ferriteglycidyl methacrylate (FG) beads (epoxy beads: TAS8848N1110) were from Tamagawa Seiki (Tokyo, Japan). 4′-hydroxy flurbiprofen was obtained from Toronto Research Chemicals (Toronto, ON, Canada).

### Measurement of chaperone activity using α-lactalbumin aggregates

Chaperone activity was measured as described previously (Huang *et al*, 2000; Li *et al*, 2001; Kubota *et al*, 2006). Aggregation was monitored in the presence or absence of reagents such as sodium 4-phenylbutyrate (4-PBA), flurbiprofen, aspirin, ibuprofen, and meloxicam by measuring turbidity at 488 nm using a VERSAmax microplate reader (Molecular Devices, Sunnyvale, CA, USA).

### Measurement of chaperone activity based on heat-induced aggregation of lysozymes

The effect of flurbiprofen on the heat-induced aggregation of lysozymes was measured as described previously with minor modifications (Kudou *et al*, 2003). In the pilot study, we confirmed the inhibition of aggregated lysozymes by adding 50 mM arginine, which was used as a positive control (Kudou *et al*, 2003) (supplementary Fig S10). Lysozyme was dissolved in phosphate buffer and mixed with flurbiprofen (dissolved in DMSO). The final concentrations of lysozyme and flurbiprofen were 1 mg/ml and 30 mM, respectively. Samples were then heated at 98°C for 10 min. Twenty minutes after the samples had stood at 25°C, aggregated lysozymes were separated by centrifugation at 15 000 g for 20 min. The concentration of soluble protein was then measured using the BCA method. Data are presented as the ratio of the concentration of lysozyme in a heated state to that in a non-heated state.

### Measurement of chaperone activity based on heat-induced aggregation of ALDH2

ALDH2 was dissolved in phosphate buffer and mixed with flurbiprofen (dissolved in DMSO). The final concentrations of Aldh2 and flurbiprofen were 0.2 mg/ml and 30 mM, respectively. Samples were then heated at 70°C for 10 min. Twenty minutes after the samples had stood at 25°C, aggregated ALDH2 was separated by centrifugation at 14 000 rpm for 20 min. The concentration of soluble protein was then measured using the BCA method.

### Dynamic light scattering

Dynamic light scattering (DLS) measurements were performed using optics composed of a 50 mW argon ion laser at a wavelength of 488 nm (Melles Griot, Tokyo, Japan), a photon counting module (Hamamatsu photonics, Hamamatsu, Japan), and a correlator board (ALV, Langen, Germany). Measurements were conducted at room temperature (27°C) at a detection angle of 45°. Autocorrelations were analyzed by a multi-tau regularized-fit procedure to obtain the distribution of decay rates. The hydrodynamic radius (*R*_h_) was then calculated using the viscosity of water, 0.85 mPa s. Lysozyme (Code 100940; Seikagaku, Tokyo, Japan, 10 mg/ml) and flurbiprofen (50 mM) were dissolved in H_2_O containing 100 mM NaOH and the pH was adjusted to 12.4. Samples were filtered with a 0.1 μm filter (Acrodisc syringe filter) and directly injected into an optical glass cell. A DLS measurement was performed before the samples were heated (before heating). The samples were then heated at 42°C for 5 min, maintained at room temperature for 20 min, and another DLS measurement was performed (after heating).

### Cell culture

HEK293 cells were maintained in Dulbecco's modified Eagle's medium with 10% (v/v) heat-inactivated fetal calf serum, 100 units/ml penicillin G, and 100 mg/ml streptomycin. Human neuroblastoma SH-SY5Y and SH-SY5Y Ob-Rb cells were maintained in Dulbecco's modified Eagle's medium supplemented with 10% (v/v) heat-inactivated fetal calf serum. All cultured cells were kept at 37°C in 5% CO_2_/95% air. To analyze leptin resistance, SH-SY5Y-Ob-Rb cells were exposed to ER stress (tunicamycin, Tm: 1 μg/ml; or brefeldin A, Bre: 0.1 μg/ml) in the presence or absence of flurbiprofen for 4 h and then moved to fresh medium containing flurbiprofen or vehicle. The leptin (0.5 μg/ml, 15 min)-induced phosphorylation of STAT3 was analyzed 4 h after the treatments by Western blotting or immunohistochemistry. To analyze UPR, SH-SY5Y cells were treated with flurbiprofen (Flu: 100 μM) in the presence or absence of tunicamycin (Tm: 0.5 μg/ml, 1 h). The medium was then replaced with fresh medium containing flurbiprofen or vehicle (4 h) and the activation of UPR was then analyzed.

### Generation of Ob-Rb leptin receptor-transfected cells (SH-SY5Y Ob-Rb cells)

The human Ob-Rb leptin receptor construct was a gift from Genetech Inc. The construct was transfected into SH-SY5Y cells using LipofectAMINE PLUS Reagent (Life Technologies Inc., Carlsbad, CA, USA) according to the manufacturer's instructions. After the transfection, stable transfectants were obtained by selection with the antibiotic G418.

### Lactate dehydrogenase (LDH) leakage assay

The viability of cells was estimated by measuring LDH leakage with a cytotoxicity detection kit (Roche Molecular Biochemical, Basel, Switzerland) according to the manufacturer's directions. LDH activity was measured as optimal density at 492 nm.

### Crystal violet assay

PBS-washed SH-SY5Y cells (35-mm dish) were stained with 1 ml of 0.1% crystal violet for 20 min at room temperature, washed several times with water, and dried. Stained cells were solubilized with 800 μl of 0.5% SDS and absorbance was measured at 590 nm.

### Measurement of aldehyde dehydrogenase activity

HEK293-Ob-Rb cells were harvested and incubated with or without flurbiprofen (100 μM, 60 min). Activity was measured using the aldehyde dehydrogenase activity colorimetric assay kit (BioVision, Milpitas, CA, USA) according to the manufacturer's directions.

### Gene expression analysis

Total RNA was isolated using TriPure Isolation Reagent (Roche Molecular Biochemical). cDNA was synthesized from 2 μg of total RNA by reverse transcription using 25 U of Superscript Reverse Transcriptase (Life Technologies Inc., Carlsbad, CA, USA) and 0.25 μg of Oligo(dt)12-18 primer (Invitrogen) in a 20-μl reaction mixture containing First-Strand Buffer (Invitrogen), 1 mM of dNTP mix, 10 mM of DTT, and 20 U of RNaseOUT Recombinant Ribonuclease Inhibitor (Invitrogen). Total RNA and the Oligo(dt) 12–18 primer were pre-incubated at 70°C for 10 min prior to reverse transcription. After incubation for 1.5 h at 46°C, the reaction was terminated by incubating samples for 15 min at 70°C. Regarding PCR amplification, 1.2 μl of cDNA was added to 10.8 μl of a reaction mix containing 0.2 μM of each primer, 0.2 mM of dNTP mix, 0.6 U of Taq polymerase (Roche Diagnostics, Basel, Switzerland), and reaction buffer. PCR was performed in a DNA Thermal Cycler (MJ Research, Waltham, MA, USA, PTC-220). CHOP; upstream, 5′-GCA CCT CCC AGA GCC CTC ACT CTC C-3′, and downstream, 5′-GTC TAC TCC AAG CCT TCC CCC TGC G-3′, XBP-1; upstream, 5′-CAG CAC TCA GAC TAC GTG CA-3′, and downstream, 5′-CAG AGG TGC ACG TAG TCT GA-3′, HERP; upstream, 5′- TGC ACC TGC TCC AGC CCC TA-3′ and downstream, 5′-GGG CTG GTC TGC TCG CCA TC-3′, GAPDH; upstream, 5′-AAA CCC ATC ACC ATC TTC CAG-3′ and downstream, 5′-AGG GGC CAT CCA CAG TCT TCT-3′. The PCR products (10 μl) were resolved by electrophoresis in an 8% polyacrylamide gel in TBE buffer. The gel was stained with ethidium bromide and photographed under ultraviolet light. The density of bands was measured using Image J 1.37v (Wayne Rasband, NIH, Bethesda, MD, USA) software.

### Immunohistochemistry

Cells were fixed with methanol for 10 min at −20°C. After being washed with PBS, the cells were incubated with 10% fetal calf serum at 37°C for 1 h, and allowed to react with an anti-p-STAT3 (Cell Signaling, Danvers, MA, USA; diluted to 1:50) antibody at 4°C overnight. The cells were then incubated with an anti-rabbit immunoglobulin G antibody conjugated with Alexa 488 (1:2000) at 37°C for 1 h. PI staining was performed by incubating cells with PI (1 μg/ml) for 5 min. The cells were visualized using confocal laser scanning microscopy.

### Animals

Adult male C57BL/6 Cr Slc mice were obtained from SLC (Hamamatsu, Japan). Mice were maintained in our animal facility at 22–24°C under a constant day-night rhythm and given food and water, *ad libitum*. They were fed either a normal chow diet (NCD: D12450B; Research Diets, NJ) or a high-fat diet (HFD: D12492; Research Diets, New Brunswick, NJ, USA). The NCD and HFD contained 10 kcal% fat and 60 kcal% fat, respectively. Flurbiprofen was dissolved in sterilized water containing NaOH and mixed into the drinking water. The concentrations of flurbiprofen and other NSAIDs were estimated based on the average amount of water intake to ensure that the total intake of the drug was the desired dosage. All animal experiments were conducted in accordance with the NIH Guide for Care and Use of Laboratory Animals and approved by the Animal Care and Use Committee at Hiroshima University.

### ob/ob mice

ob/ob mice were obtained from Japan SLC. Nine-week-old female ob/ob mice were housed individually before this experiment. Three days after the isolation, saline (5 ml/kg) was injected intraperitoneally (i.p.) for 3 days, and then leptin (1 mg/kg, i.p.) and/or flurbiprofen (10 mg/kg, i.p.) were injected for 6 days. All treatments were conducted once a day at 18:00.

### Computed tomography

Visceral and subcutaneous fat contents were measured using Latheta V3.00 (ALOKA CO. LTD., Tokyo Japan). Mice were fed the NCD or HFD with or without flurbiprofen (10 mg/kg/day) for 7 weeks, then isolated for 6 days, and injected with 0.9% NaCl for 3 days. After that, they were injected with leptin (0.5 mg/kg; twice in a day) for 3 days and euthanized using CO_2_ gas. Their fat contents were measured.

### Body weight

Body weight was measured once a week at 16:00–17:00.

### Measurement of leptin levels

Blood samples (including EDTA) were taken from animals by decapitation and were centrifuged (4°C, 700 g, 15 min) to obtain plasma samples. Plasma leptin levels were measured by ELISA according to the manufacturer's guidelines (R'D Systems).

### Silver staining

Eluted samples were electrophoresed and analyzed by silver staining using the Silver Stain MS kit (Wako, Japan) according to the manufacturer's directions. The stained 50-KDa bands were analyzed by Nano LC MS/MS. This analysis was conducted at Japan Bio Services Co., LTD. (Saitama, Japan).

### Western blot analysis

Western blot analysis was performed as described previously (Hosoi *et al*, 2010). The membranes were incubated with anti-phospho STAT3 (Tyr705: Cell Signaling; 1:1,000), anti-STAT3 (Santa Cruz, Santa Cruz, CA, USA; 1:1,000), anti-ALDH2 (Santa Cruz; 1:1000), and anti-ALDH1B1 (Santa Cruz; 1:1,000) antibodies, followed by an anti-horseradish peroxidase-linked antibody. Peroxidase was detected using an enhanced chemiluminescence system (GE Healthcare, Fairfield, CT, USA).

### Protein affinity purification with flurbiprofen-immobilized beads

Epoxylated magnetic FG beads (2.5 mg), were incubated with 4′-hydroxy flurbiprofen in N,N-dimethylformamide (DMF) and potassium carbonate for 24 h at 60°C (supplementary Fig S5). After several washes with DMF, the beads were suspended with 50% MeOH and stored at 4°C prior to use. Flurbiprofen-immobilized beads (0.5 mg) were washed with 100 mM KCl buffer containing 20 mM HEPES-NaOH (pH7.9), 100 mM KCl, 1 mM MgCl_2_, 0.2 mM CaCl_2_, 0.2 mM EDTA, 10% (v/v) glycerol, 0.1% NP-40, 1 mM DTT, and 0.2 mM PMSF. Sample were then equilibrated with lysis buffer containing 10 mM HEPES-NaOH (pH 7.5), 150 mM NaCl, 1 mM EGTA, 1 mM Na_3_VO_4_, 10 mM NaF, 10 μg/ml of aprotinin, 10 μg/ml of leupeptin, 1 mM phenylmethylsulfonyl fluoride (PMSF), and 1% NP-40. Cell lysates or tissue extracts (0.6 mg) prepared from C57BL/6 Cr Slc mice were incubated with the beads for 4 h at 4°C. The beads were washed three times with 100 mM KCl buffer and eluted with 1 M KCl buffer (20 mM HEPES-NaOH (pH 7.9), 1M KCl, 1 mM MgCl_2_, 0.2 mM CaCl_2_, 0.2 mM EDTA, 10% (v/v) glycerol, 0.1% NP-40, 1 mM DTT, and 0.2 mM PMSF). The bound proteins were eluted by boiling samples with Laemmli buffer. Bound proteins for the drug elution experiments were eluted with 100 mM KCl buffer containing 1–5 mM flurbiprofen. A total of 0.2–1.0 mM flurbiprofen was added to extracts before incubation with the beads for the competitive inhibition experiments. FG beads were incubated with 0.1 μg of recombinant ALDH2 in a BSA (9 μg/μl) solution for the direct interaction experiments.

### RNAi experiments

Transient transfections of siRNA were performed in SH-SY5Y cells, HEK293-Ob-Rb cells, and HEK293T cells. Lipofectamine RNAiMAX (Life Technologies) was used to transfect siRNA according to the manufacturer's directions. We used the following siRNA sequences for SH-SY5Y and HEK293T cells: human ALDH2: 5′-CAG AUC AUU CCG UGG AAU UdTdT-3′; human ALDH1B1: 5′-GCU ACA UCC AGC UUG GCC A dTdT-3′, and MISSION siRNA Universal Negative Control (SIGMA; SIC-001) for the control siRNA transfection. We used Silencer(R) Select Validated siRNAs (Life Technologies, siRNA IDs for ALDH2 and ALDH1B1: s1239, s1240, s1245, s1246) or Silencer Select Negative Control siRNA #1 (Life technologies) for HEK293-Ob-Rb cells. Cells were harvested 72–96 h after transfection.

### Statistics

Results are expressed as the mean ± s.e. Statistical analyses were performed using the Student's *t*-test or Paired *t*-test.

**The paper explained**

**Problem**

Obesity has become a serious global health concern because it is the main risk factor for several diseases such as diabetes, nonalcoholic steatohepatitis (NASH), hypertension, cardiovascular disease, and cancer. Several anti-obesity drugs have been developed. However, because most treatments are based on symptoms, efficient fundamental treatments are still needed. Therefore, the goal of this study was to identify a novel type of drug for the fundamental treatment of obesity.

**Results**

Protein misfolding may be involved in the development of obesity. Therefore, the attenuation of protein misfolding may represent a fundamental treatment. Chemically reducing the accumulation of unfolded proteins may be a novel form of treatment. In the present study, we discovered a drug that attenuated protein misfolding, thereby preventing obesity. We demonstrated that flurbiprofen, a nonsteroidal anti-inflammatory drug (NSAID), inhibited protein aggregation and attenuated obesity in mice. By reducing protein aggregation, flurbiprofen attenuated endoplasmic reticulum (ER) stress and the subsequent development of leptin resistance, characterized by insensitivity to the anti-obesity hormone leptin. The other NSAIDs tested had no such effect, which suggested that flurbiprofen has the unique pharmacological property of attenuating obesity differently from the other NSAIDs. To clarify the mechanism responsible for this unique effect, we identified the target protein interacting with flurbiprofen, aldehyde dehydrogenase. The knock down of aldehyde dehydrogenase enhanced ER stress-induced cell death; therefore, interactions with this protein may play an important role in preventing ER stress and the subsequent development of obesity.

**Impact**

Our findings are innovative as they revealed a good example of a drug for the fundamental treatment of obesity. Several anti-obesity drugs have serious side-effects. On the other hand, flurbiprofen is a classic drug that has been used safely for decades. Therefore, the present results may offer a safe treatment for metabolic syndrome.

## For more information

website:http://www.mccanet.nl
