# Supplementary material for: Flurbiprofen ameliorated obesity by attenuating leptin resistance induced by endoplasmic reticulum stress
Source: EMBO Mol Med. 2014 Jan 14;6(3):335–46. doi: 10.1002/emmm.201303227 (PMC3958308; doi:10.1002/emmm.201303227)
Supplement: Supplementary file 1 [file emmm0006-0335-sd1.pdf]

EMBO Molecular Medicine

Flurbiprofen ameliorated obesity by attenuating leptin resistance induced by endoplasmic reticulum stress

Toru Hosoi, Rie Yamaguchi, Kikuko Noji, Suguru Matsuo, Sachiko Baba, Keisuke Toyoda, Takahiro Suezawa, Takaaki Kayano, Shinpei Tanaka, and Koichiro Ozawa

Corresponding author: Toru Hosoi, Hiroshima university

Review timeline:

Submission date:	25 June 2013
Accepted:	21 November 2013

Editor: Céline Carret

Transaction Report:

No Peer Review Process File is available with this article, as the authors have chosen not to make the review process public in this case.

© EMBO
