# Supplementary figures and images for: Flurbiprofen ameliorated obesity by attenuating leptin resistance induced by endoplasmic reticulum stress
Source: EMBO Mol Med. 2014 Jan 14;6(3):335–46. doi: 10.1002/emmm.201303227 (PMC3958308; doi:10.1002/emmm.201303227)

**Fig. 3A**

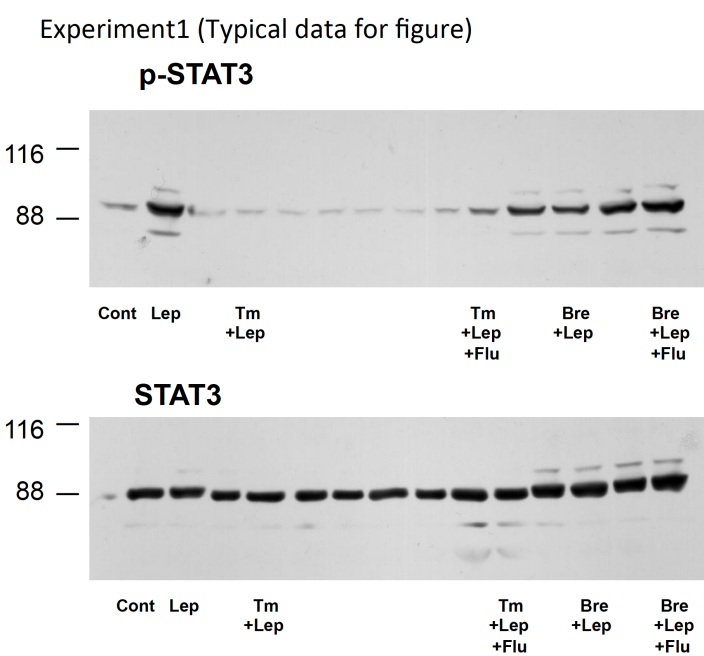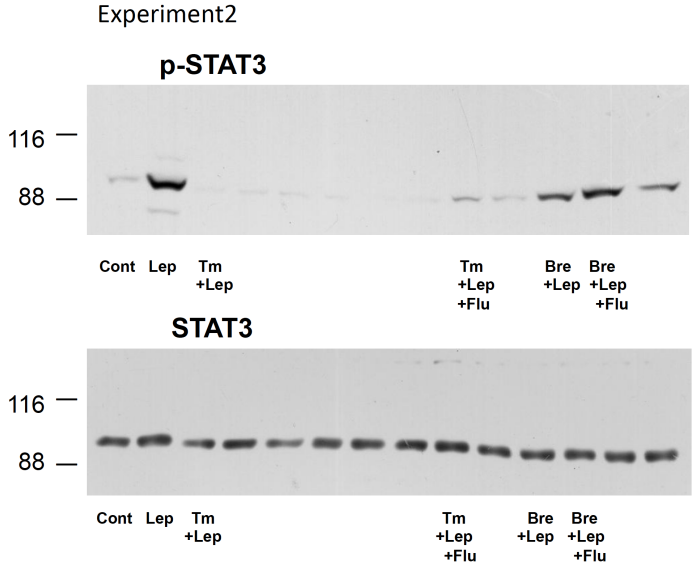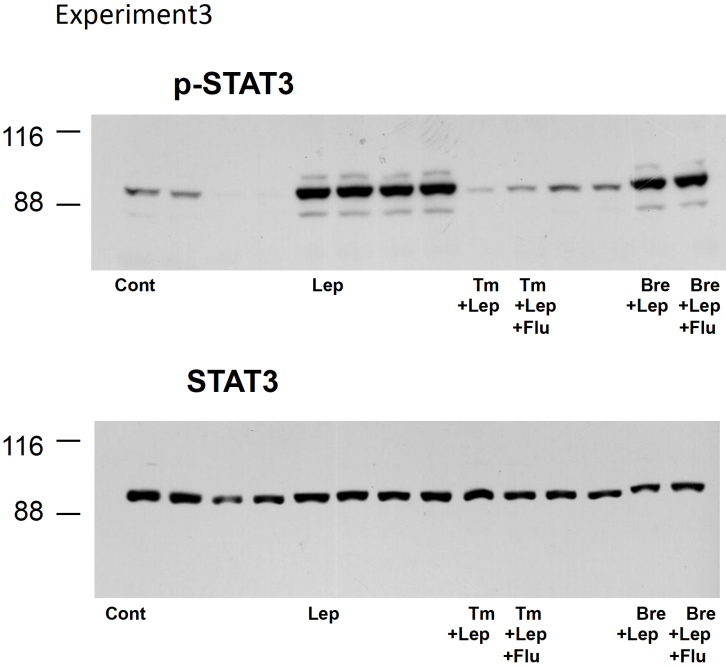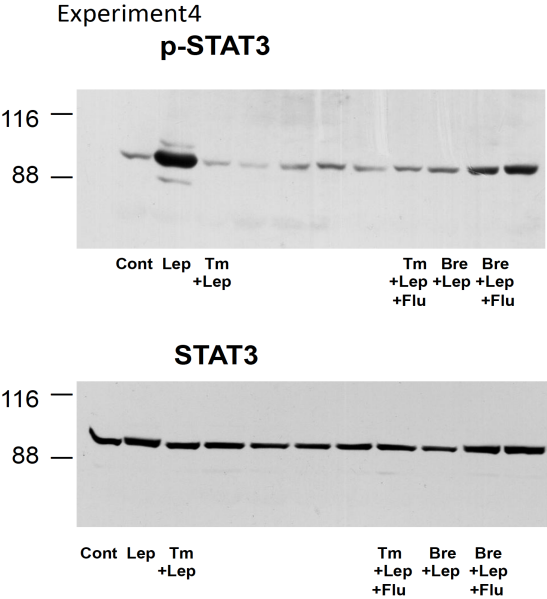

Supplement: Supplementary file 2 [file emmm0006-0335-sd2.pdf]

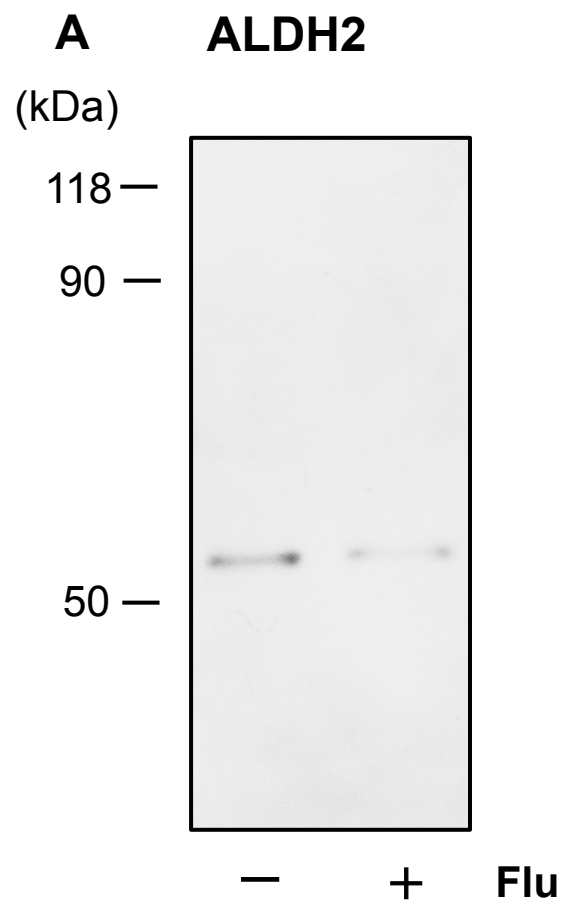

Fig.6

Supplement: Supplementary file 4 [file emmm0006-0335-sd4.pdf]

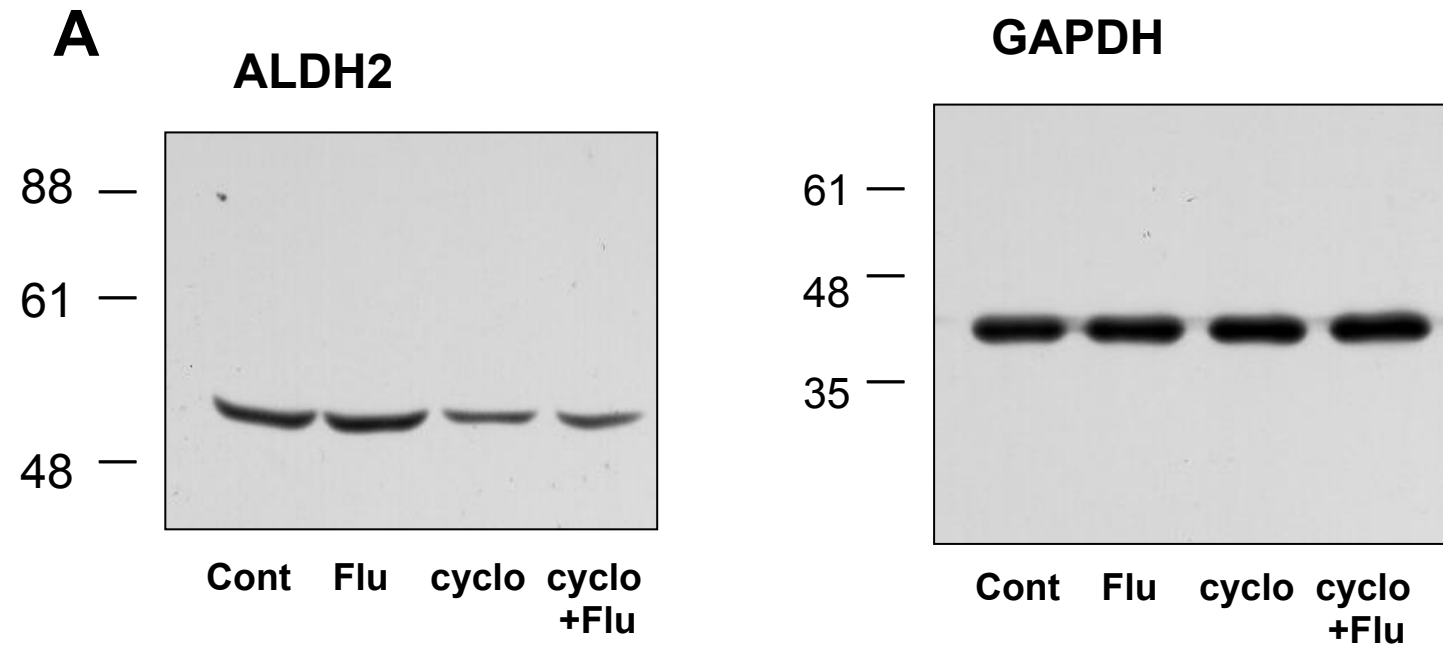

Fig.7

Supplement: Supplementary file 5 [file emmm0006-0335-sd5.pdf]

Fig. 8A

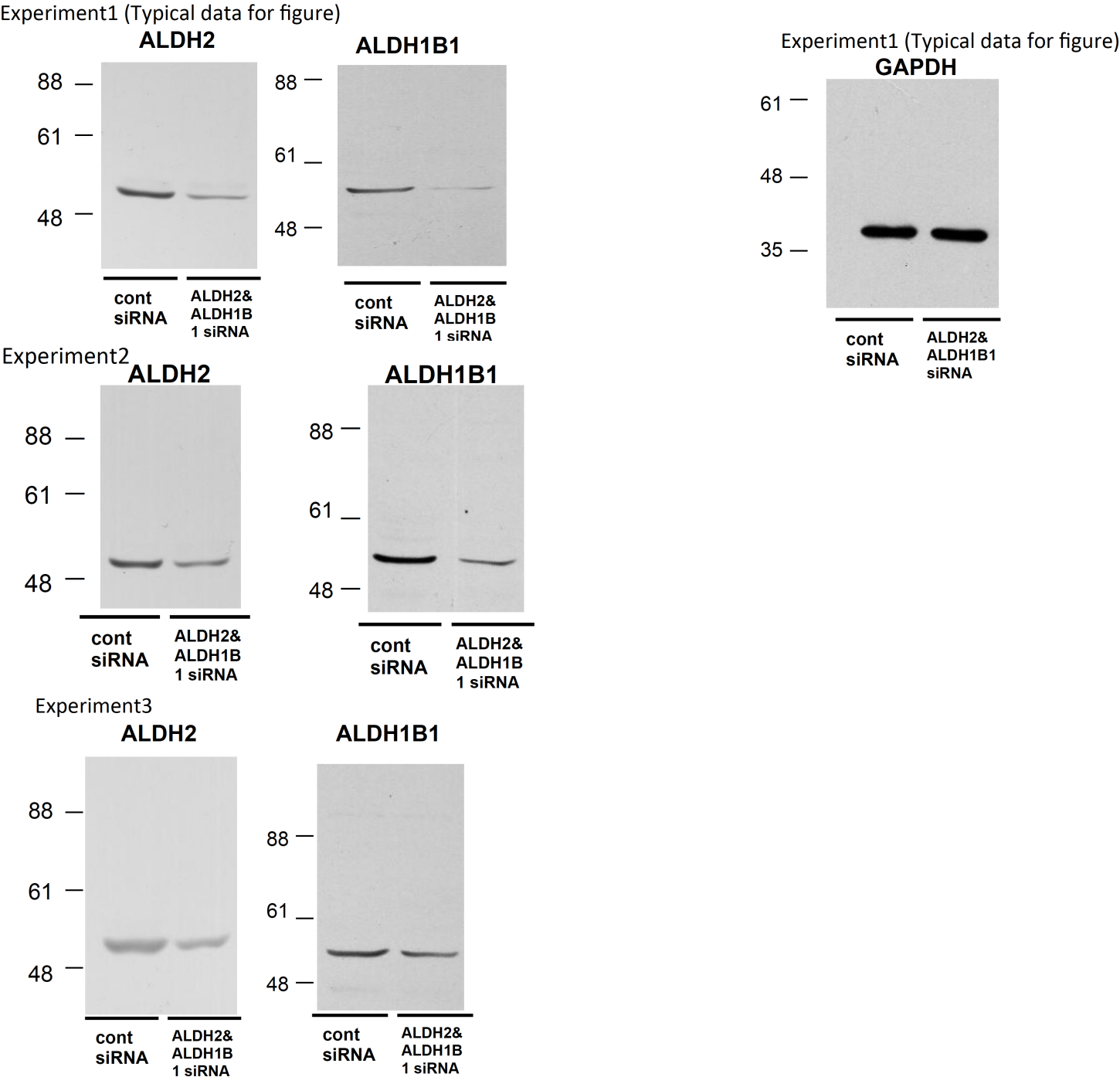

Fig. 8B

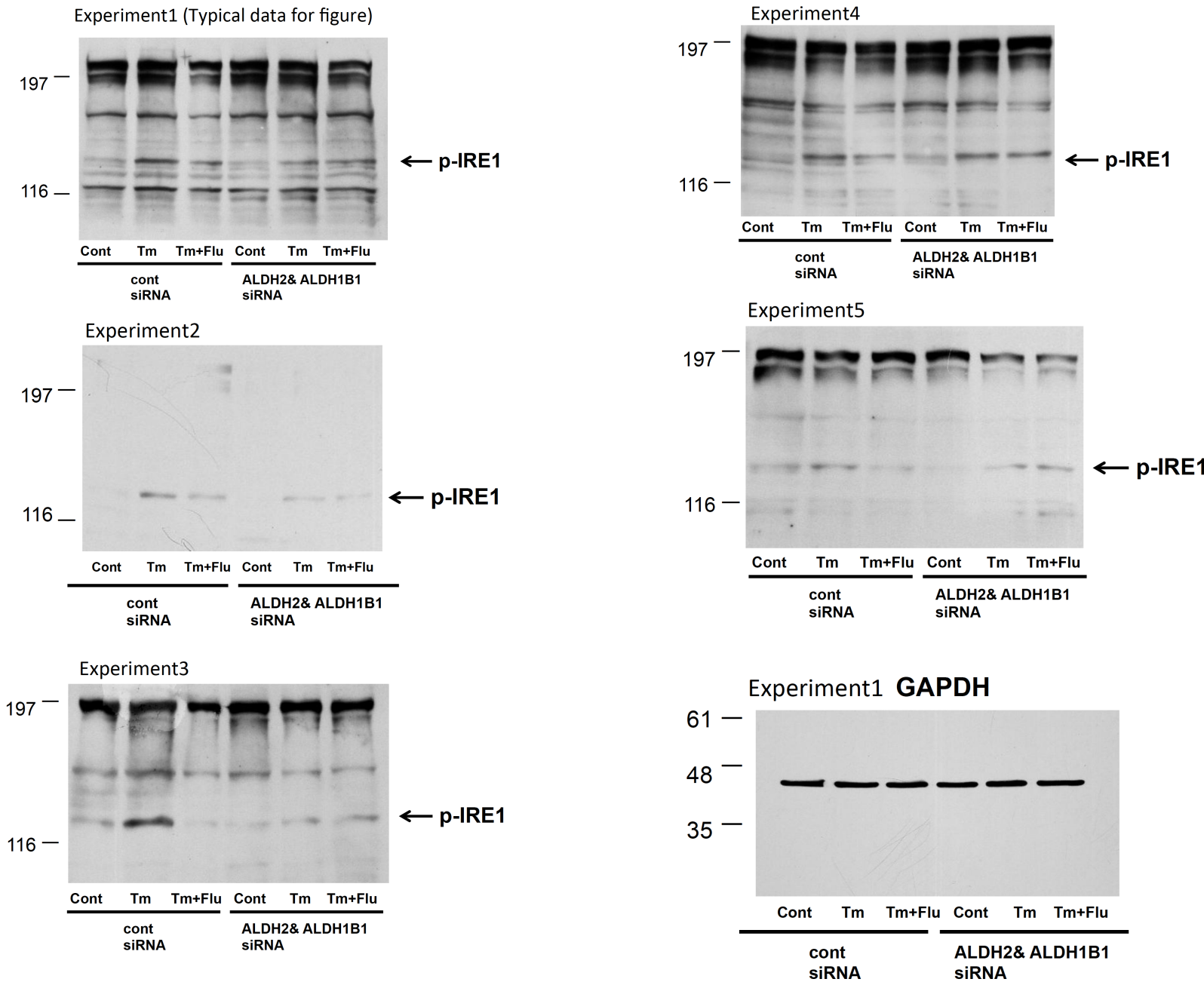

Fig. 8C

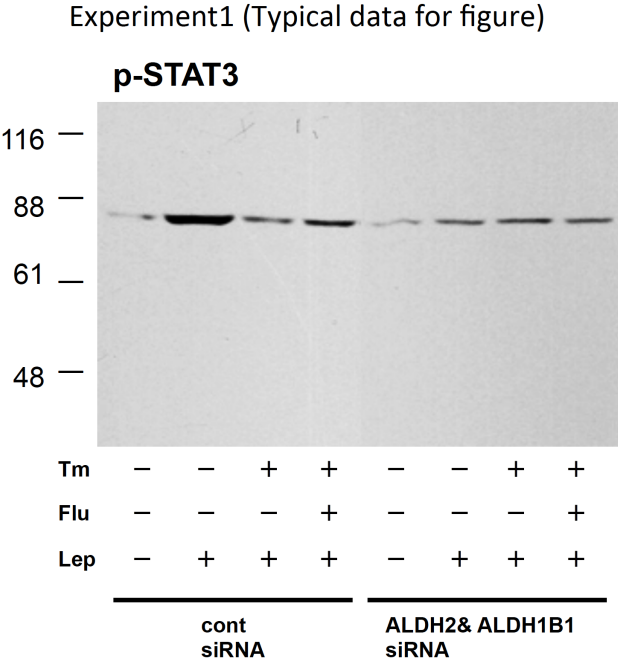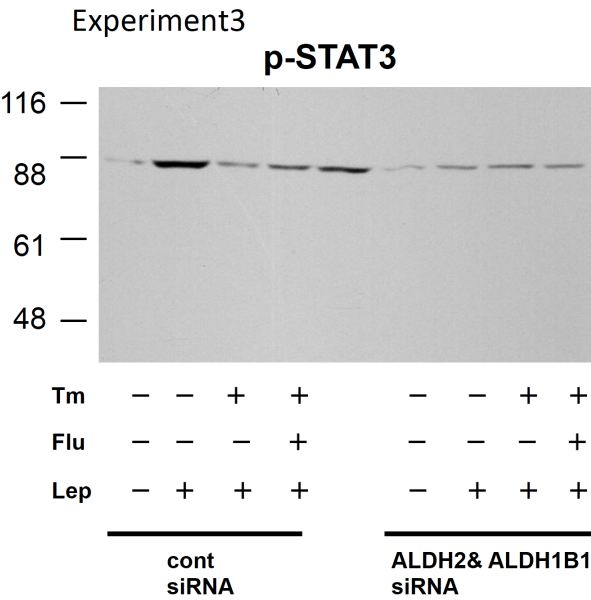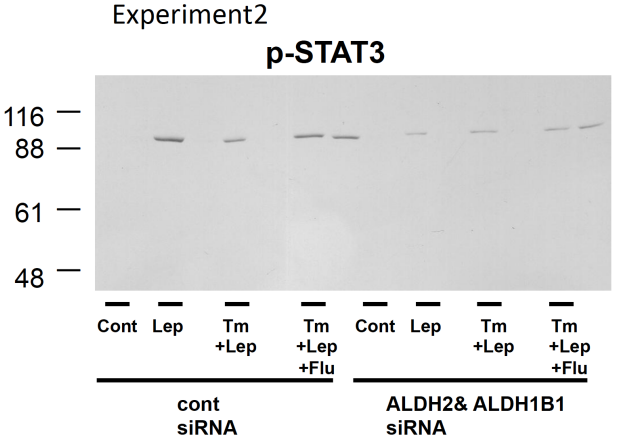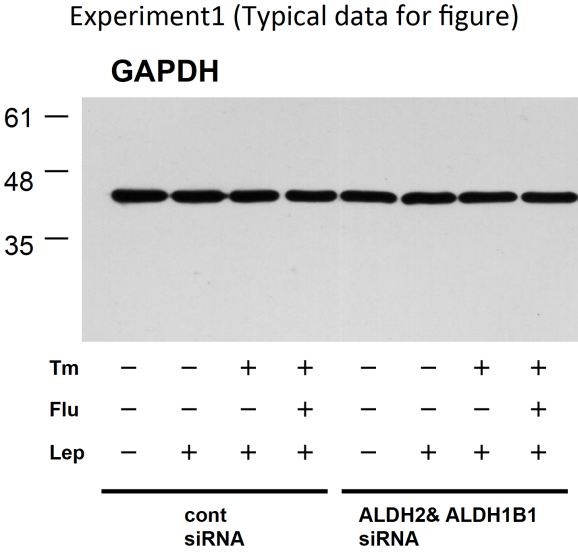

Supplement: Supplementary file 6 [file emmm0006-0335-sd6.pdf]
