# Supplementary material for: Flurbiprofen ameliorated obesity by attenuating leptin resistance induced by endoplasmic reticulum stress
Source: EMBO Mol Med. 2014 Jan 14;6(3):335–46. doi: 10.1002/emmm.201303227 (PMC3958308; doi:10.1002/emmm.201303227)

Fig. 5A

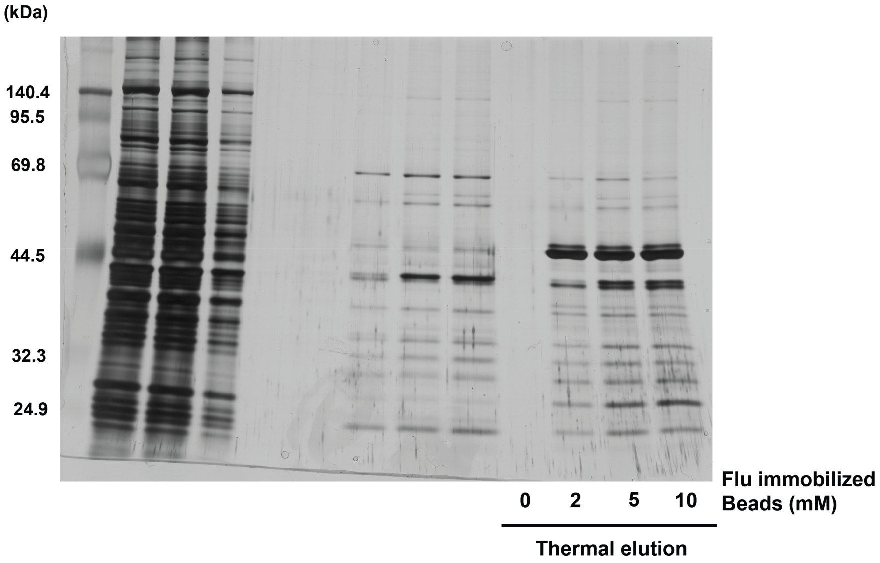

Fig. 5C

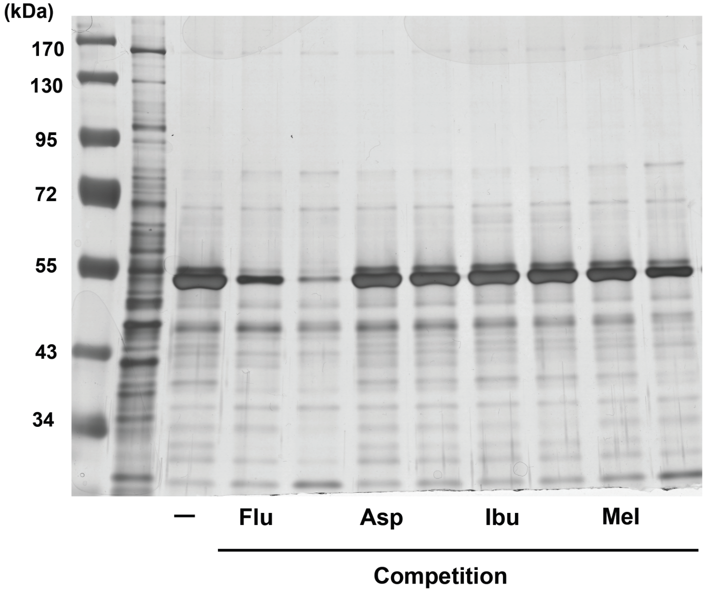

Fig. 5B

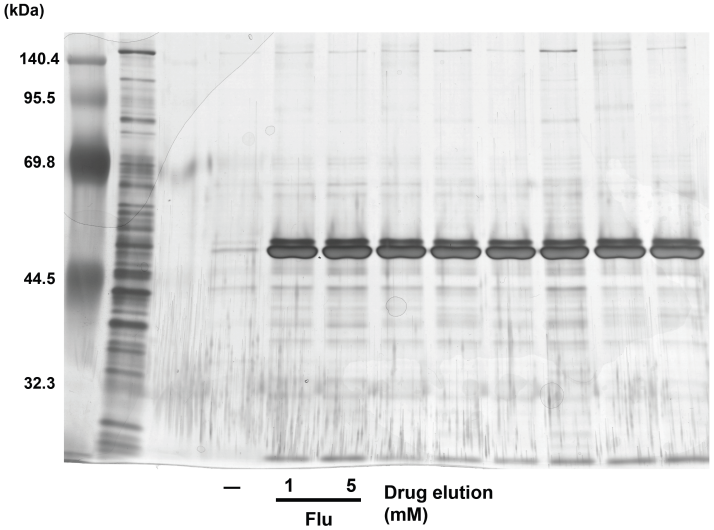

Fig. 5D

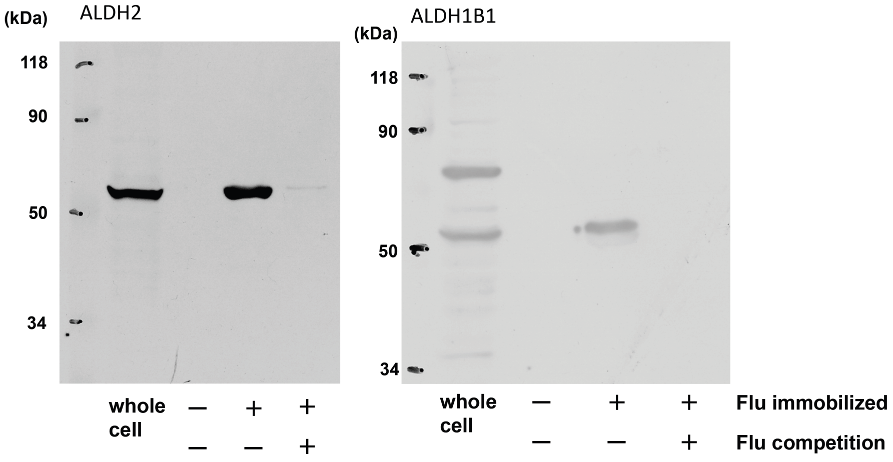

Fig. 5E (ALDH2)

ALDH2
experiment 1
(Typical data for figure)

(kDa)

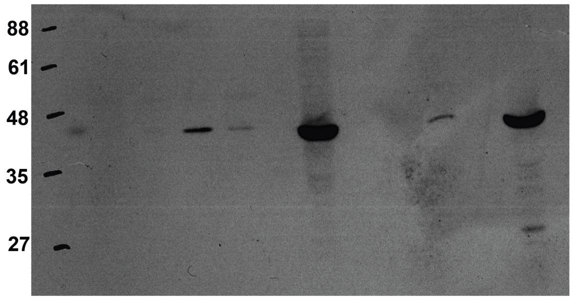

— — + +

cont. siRNA | cont. siRNA

ALDH2& ALDH1B1 siRNA | ALDH2& ALDH1B1 siRNA

(kDa) experiment 2

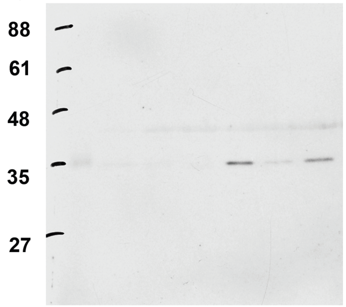

— — + +

cont. siRNA | ALDH2& ALDH1B1 siRNA | cont. siRNA | ALDH2& ALDH1B1 siRNA

Fig. 5E (ALDH1B1)

ALDH1B1
experiment 1
(Typical data for figure)

(kDa)

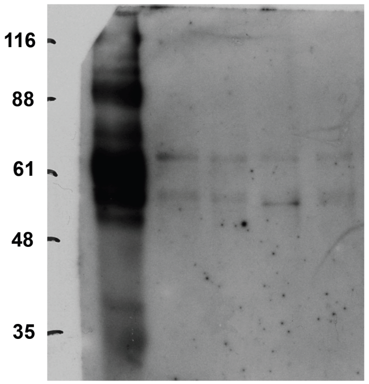

— — + +

cont. siRNA | cont. siRNA

ALDH2& ALDH1B1 siRNA | ALDH2& ALDH1B1 siRNA

(kDa) experiment2

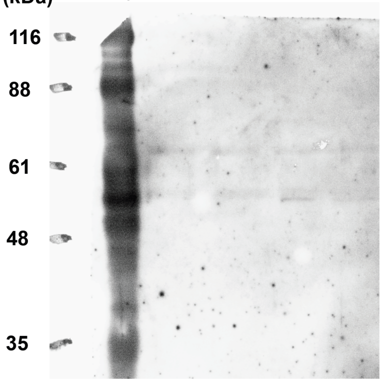

— — + +

cont. siRNA | cont. siRNA

ALDH2& ALDH1B1 siRNA | ALDH2& ALDH1B1 siRNA

Fig. 5F

Experiment 1
(Typical data for figure)

(kDa)

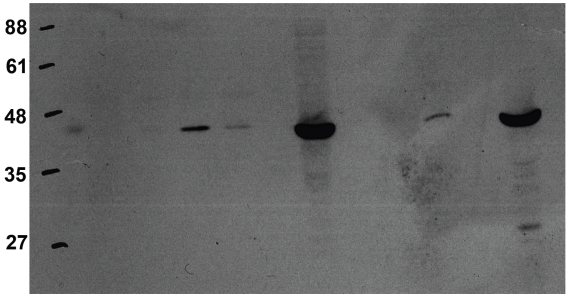

— + **Flu immobilized**

(kDa) experiment 2

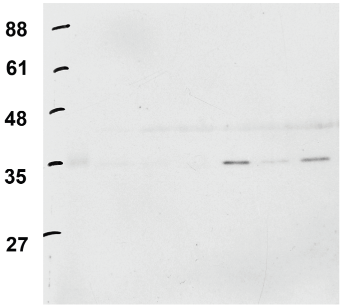

— + **Flu immobilized**

Fig. 5G

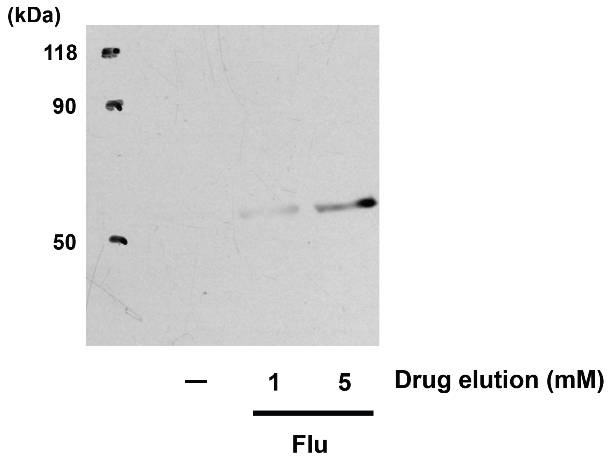

Fig. 5I

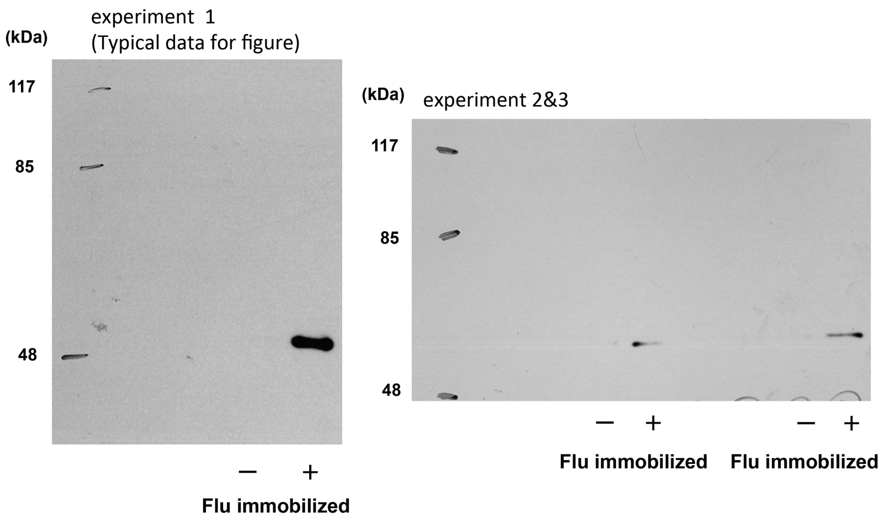

Fig. 5H

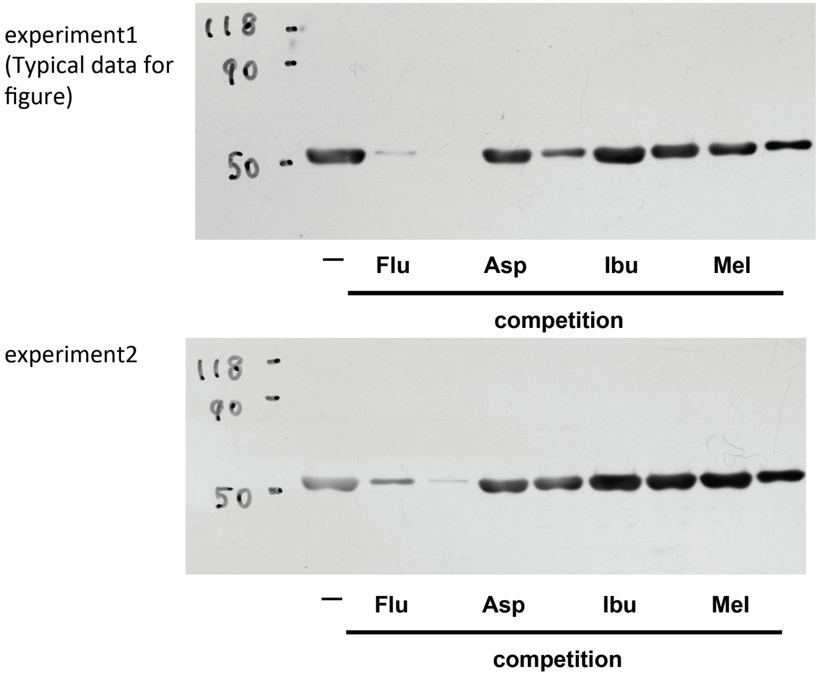

Supplement: Supplementary file 3 [file emmm0006-0335-sd3.pdf]
