# Supplementary material for: Flurbiprofen ameliorated obesity by attenuating leptin resistance induced by endoplasmic reticulum stress
Source: EMBO Mol Med. 2014 Jan 14;6(3):335–46. doi: 10.1002/emmm.201303227 (PMC3958308; doi:10.1002/emmm.201303227)
Supplement: Supplementary file 7 [file emmm0006-0335-sd7.pdf]

Supporting Information

Flurbiprofen ameliorated obesity by attenuating leptin resistance induced by endoplasmic reticulum stress

Toru Hosoi[1], Rie Yamaguchi[1], Kikuko Noji[1], Suguru Matsuo[1], Sachiko Baba[1], Keisuke Toyoda[1], Takahiro Suezawa[1], Takaaki Kayano[1], Shinpei Tanaka[2] and Koichiro Ozawa[1]

[1]Department of Pharmacotherapy, Graduate School of Biomedical and Health Sciences, Hiroshima University, 1-2-3 Kasumi, Minami-ku, Hiroshima 734-8553, Japan

[2]School of Integrated Arts & Sciences, Hiroshima University, 1-7-1 Kagamiyama, Higashi-Hiroshima 739-8521, Japan.

Table of contents:

Supplementary Figure. S1 (page 2)
Flurbiprofen inhibited ER stress-induced cell death.

Supplementary Figure. S2 (page 2)
Circulating leptin levels were normalized by flurbiprofen treatment.

Supplementary Figure. S3 (page 3)
Effect of flurbiprofen on leptin-induced attenuation of food intake.

Supplementary Figure. S4 (page 3)
CT scan analysis of adipose tissue of flurbiprofen-treated mice.

Supplementary Figure. S5 (page 4)
Flurbiprofen did not influence mice locomotor activities.

Supplementary Figure. S6 (page 4)
Fulrbiprofen did not affect body length.

Supplementary Figure. S7 (page 5)
Preparation of flurbiprofen-immobilized beads.

Supplementary Figure. S8 (page 5)
Flurbiprofen-bound proteins were analyzed in other tissue lysates.

Supplementary Figure. S9 (page 6)
ER stress-induced cell death was enhanced by knocking down ALDH2 and ALDH1B1.

Supplementary Figure. S10 (page 6)
Heat-induced aggregation of lysozymes was measured with or without arginine (Arg).
