# Supplementary material for: Flurbiprofen ameliorated obesity by attenuating leptin resistance induced by endoplasmic reticulum stress
Source: EMBO Mol Med. 2014 Jan 14;6(3):335–46. doi: 10.1002/emmm.201303227 (PMC3958308; doi:10.1002/emmm.201303227)
Supplement: Supplementary file 8 [file emmm0006-0335-sd8.pdf]

Supplemental figures

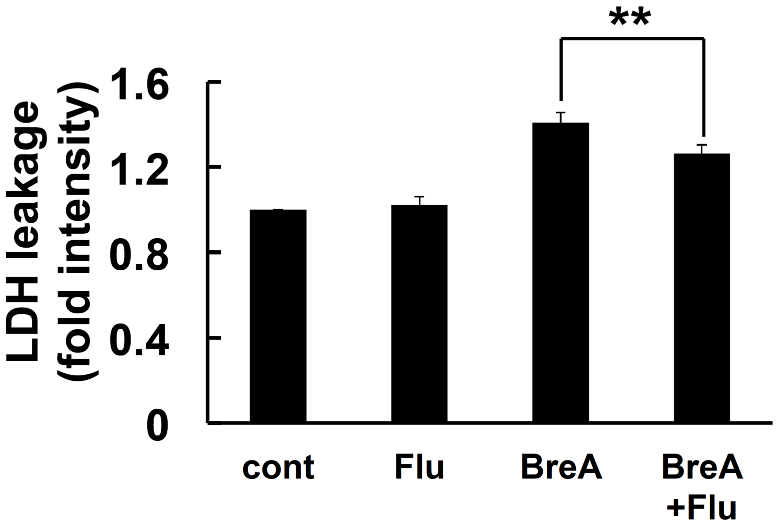

Fig. S1 Flurbiprofen inhibited ER stress-induced cell death.
SH-SY5Y cells were treated with brefeldin A (30 ng/mL) in the presence or absence of flurbiprofen (Flu) for 48 h. LDH activity was then measured as an indicator of cytotoxicity. ***P*<0.01 n=8.

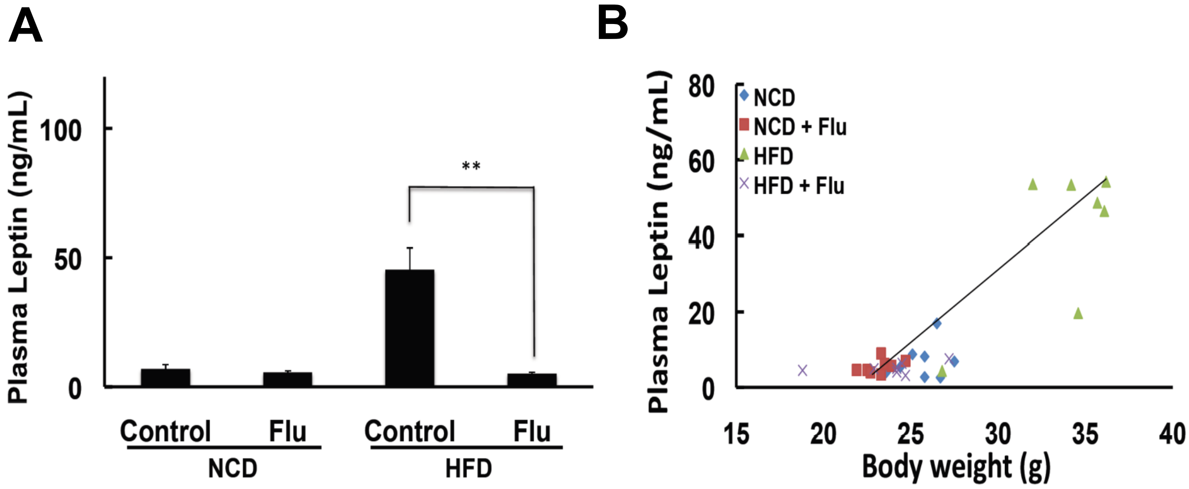

Fig. S2 Circulating leptin levels were normalized by flurbiprofen treatment
(**A**) Flurbiprofen inhibited the high-fat diet (HFD)-induced elevation of circulating leptin levels in fasted mice. Mice were fed a normal chow diet (NCD) or high-fat diet (HFD) with or without flurbiprifen (Flu) for 8 weeks. Plasma leptin levels were measured 17 h after fasting. We observed that flurbiprofen can reduce plasma leptin levels even in a fasted mice. n = 7 ~ 8 per group. ****P* < 0.001 v.s. high-fat diet. (**B**) Leptin levels were positively correlated with body weight.
