# Supplementary material for: Flurbiprofen ameliorated obesity by attenuating leptin resistance induced by endoplasmic reticulum stress
Source: EMBO Mol Med. 2014 Jan 14;6(3):335–46. doi: 10.1002/emmm.201303227 (PMC3958308; doi:10.1002/emmm.201303227)
Supplement: Supplementary file 9 [file emmm0006-0335-sd9.pdf]

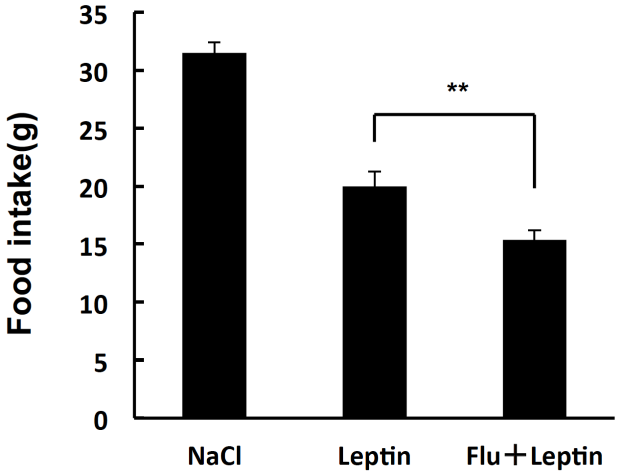

Fig. S3 Effect of flurbiprofen on leptin-induced attenuation of food intake.
ob/ob mice were treated with flurbiprofen (Flu) in combination with leptin once per day for 6 days and food intake was analyzed. Data were expressed as the total cumulative amount of food intake. Flurbiprofen significantly enhanced the effects of leptin on feeding reduction. $**P < 0.01$ v.s. leptin-treatment alone. n=9-12.

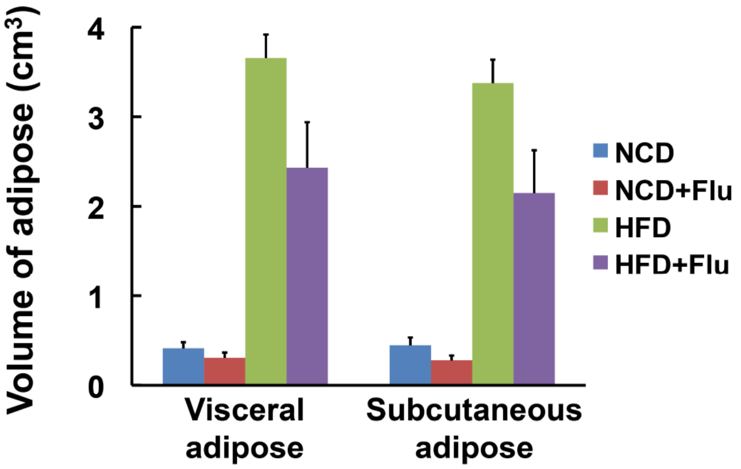

Fig. S4 CT scan analysis of adipose tissue of flurbiprofen-treated mice.
Flurbiprofen similarly inhibited the high-fat diet-induced accumulation of visceral or subcutaneous adipose tissue. n=6-8 per group.
