# Supplementary material for: Flurbiprofen ameliorated obesity by attenuating leptin resistance induced by endoplasmic reticulum stress
Source: EMBO Mol Med. 2014 Jan 14;6(3):335–46. doi: 10.1002/emmm.201303227 (PMC3958308; doi:10.1002/emmm.201303227)
Supplement: Supplementary file 10 [file emmm0006-0335-sd10.pdf]

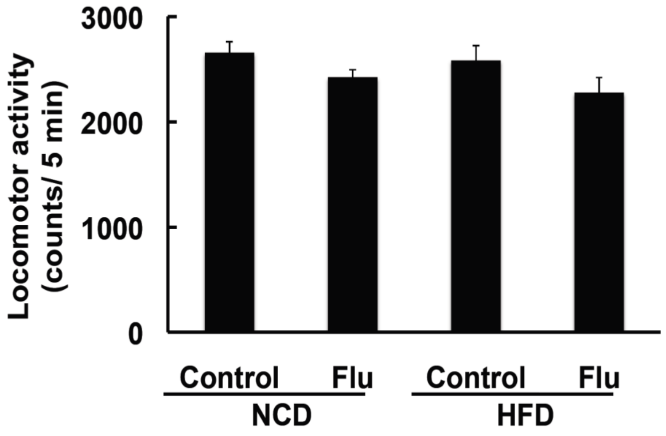

Fig. S5 Flurbiprofen did not influence mice locomotor activities.
Open field tests were used to measure locomotor activity. Total activity in 5 minutes was roughly the same among mice. n = 15-16 per group.

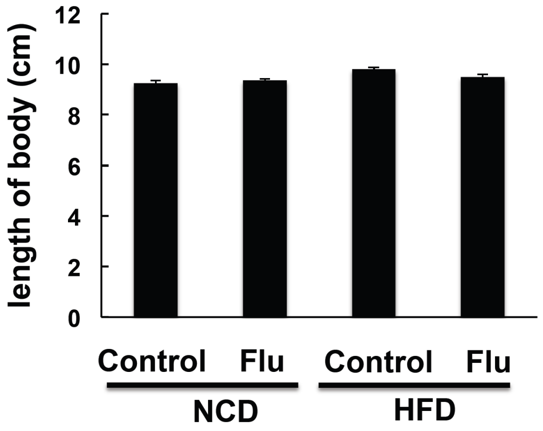

Fig.S6 Fulrbiprofen did not affect body length.
Mice were fed a normal chow diet (NCD) or high fat diet (HFD) concomitantly with or without flurbiprofen (Flu) for 8 weeks. Body length was measured 8 weeks after the treatments in each mouse. We measured body lengths from the nose to the beginning of the tail. n = 7-8 per group.
