# Supplementary material for: Flurbiprofen ameliorated obesity by attenuating leptin resistance induced by endoplasmic reticulum stress
Source: EMBO Mol Med. 2014 Jan 14;6(3):335–46. doi: 10.1002/emmm.201303227 (PMC3958308; doi:10.1002/emmm.201303227)
Supplement: Supplementary file 11 [file emmm0006-0335-sd11.pdf]

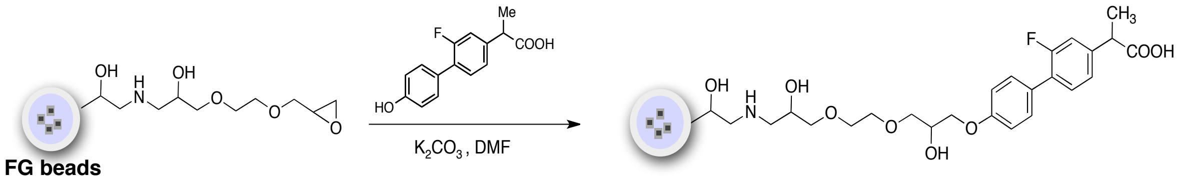

Fig. S7 Preparation of flurbiprofen-immobilized beads.
FG beads were incubated with 4’-hydroxy flurbiprofen and K_2CO_3 in DMF for 24 h at 60 °C.

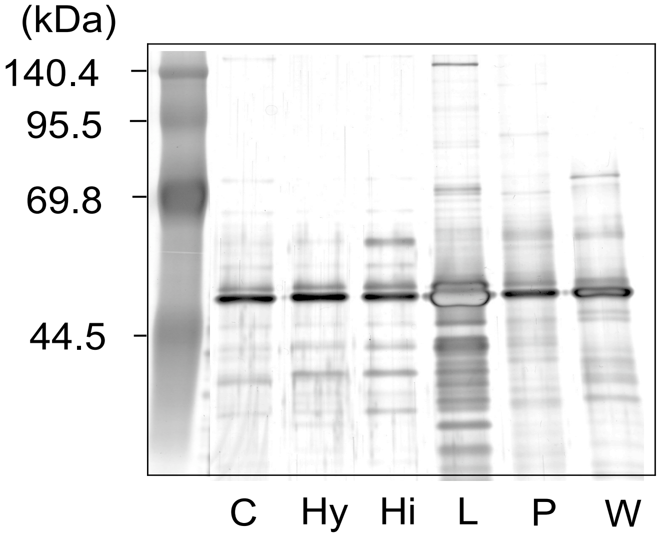

Fig. S8 Flurbiprofen-bound proteins were analyzed in other tissue lysates.
C: cerebral cortex, Hy: hypothalamus, Hi: hippocampus, L: liver, P: pancreas, W: white adipocyte.
