# Supplementary material for: Flurbiprofen ameliorated obesity by attenuating leptin resistance induced by endoplasmic reticulum stress
Source: EMBO Mol Med. 2014 Jan 14;6(3):335–46. doi: 10.1002/emmm.201303227 (PMC3958308; doi:10.1002/emmm.201303227)
Supplement: Supplementary file 12 [file emmm0006-0335-sd12.pdf]

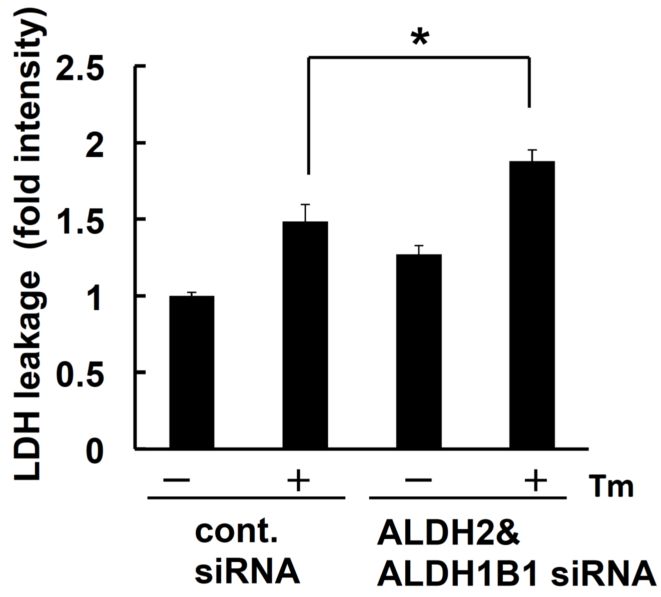

Fig. S9 ER stress-induced cell death was enhanced by knocking down ALDH2 and ALDH1B1.

SH-SY5Y cells were treated with tunicamycin (Tm: 1μg/ mL) for 24 h and LDH activity was measured as an indicator of cytotoxicity. *$P< 0.05$, n= 4. We knocked down ALDH2 and ALDH1B1 using the following siRNA sequences: human ALDH2: 5'-CUC UAU GUG GCC AAC CUG AdTdT-3'; human ALDH1B1: 5'-GGC UUA AGG CCU ACA CAG AdTdT-3'.

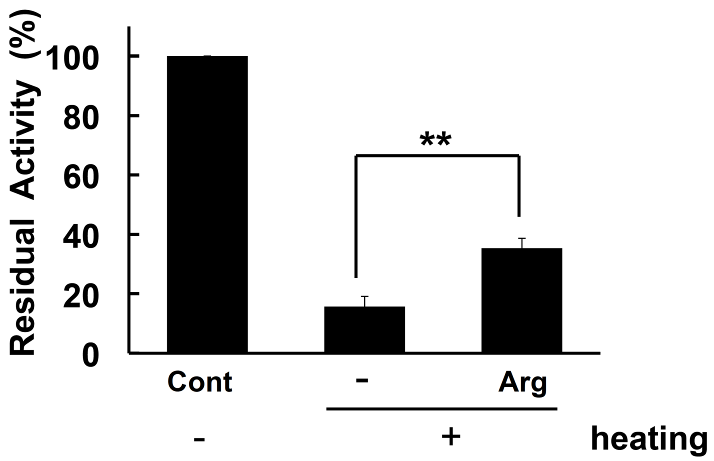

Fig. S10 Heat-induced aggregation of lysozymes was measured with or without arginine (Arg).

Arginine inhibited the heat-induced aggregation of lysozymes.
